# BIN1 Localizes the L-Type Calcium Channel to Cardiac T-Tubules

**DOI:** 10.1371/journal.pbio.1000312

**Published:** 2010-02-16

**Authors:** Ting-Ting Hong, James W. Smyth, Danchen Gao, Kevin Y. Chu, Jacob M. Vogan, Tina S. Fong, Brian C. Jensen, Henry M. Colecraft, Robin M. Shaw

**Affiliations:** 1 Cardiovascular Research Institute, University of California San Francisco, San Francisco, California, United States of America; 2 Department of Medicine, University of California San Francisco, San Francisco, California, United States of America; 3 Department of Physiology, Columbia University, New York, New York, United States of America; Massachusetts General Hospital, United States of America

## Abstract

The BAR domain protein superfamily is involved in membrane invagination and endocytosis, but its role in organizing membrane proteins has not been explored. In particular, the membrane scaffolding protein BIN1 functions to initiate T-tubule genesis in skeletal muscle cells. Constitutive knockdown of BIN1 in mice is perinatal lethal, which is associated with an induced dilated hypertrophic cardiomyopathy. However, the functional role of BIN1 in cardiomyocytes is not known. An important function of cardiac T-tubules is to allow L-type calcium channels (Cav1.2) to be in close proximity to sarcoplasmic reticulum-based ryanodine receptors to initiate the intracellular calcium transient. Efficient excitation-contraction (EC) coupling and normal cardiac contractility depend upon Cav1.2 localization to T-tubules. We hypothesized that BIN1 not only exists at cardiac T-tubules, but it also localizes Cav1.2 to these membrane structures. We report that BIN1 localizes to cardiac T-tubules and clusters there with Cav1.2. Studies involve freshly acquired human and mouse adult cardiomyocytes using complementary immunocytochemistry, electron microscopy with dual immunogold labeling, and co-immunoprecipitation. Furthermore, we use surface biotinylation and live cell confocal and total internal fluorescence microscopy imaging in cardiomyocytes and cell lines to explore delivery of Cav1.2 to BIN1 structures. We find visually and quantitatively that dynamic microtubules are tethered to membrane scaffolded by BIN1, allowing targeted delivery of Cav1.2 from the microtubules to the associated membrane. Since Cav1.2 delivery to BIN1 occurs in reductionist non-myocyte cell lines, we find that other myocyte-specific structures are not essential and there is an intrinsic relationship between microtubule-based Cav1.2 delivery and its BIN1 scaffold. In differentiated mouse cardiomyocytes, knockdown of BIN1 reduces surface Cav1.2 and delays development of the calcium transient, indicating that Cav1.2 targeting to BIN1 is functionally important to cardiac calcium signaling. We have identified that membrane-associated BIN1 not only induces membrane curvature but can direct specific antegrade delivery of microtubule-transported membrane proteins. Furthermore, this paradigm provides a microtubule and BIN1-dependent mechanism of Cav1.2 delivery to T-tubules. This novel Cav1.2 trafficking pathway should serve as an important regulatory aspect of EC coupling, affecting cardiac contractility in mammalian hearts.

## Abstract 2

Cardiac tubular-like membrane invaginations contain the membrane scaffolding protein BIN1, which tethers dynamic microtubules that deliver calcium channels directly to T-tubule membrane.

## Author Summary

Calcium plays a primary role in regulating heart function. During each heartbeat, calcium ions cross the membrane of individual cardiac muscle cells and trigger a rapid increase of calcium within the cell (called the calcium transient). Calcium causes the muscle cells to contract and determines the strength of the overall heartbeat. Each cardiac muscle cell has many small tubular-like membrane invaginations known as T-tubules where calcium channels localize, allowing calcium ions to enter and immediately encounter intracellular calcium release organelles. While this organization is well described, it is not known how calcium channels localize to T-tubule membrane. Here we show that in human and mouse heart cells, a membrane scaffolding protein known as BIN1 is localized together with calcium channels at T-tubules. Using high-resolution live cell microscopy, we found that microtubules, which are necessary for calcium channel delivery to the membrane, are also tethered by BIN1. Loss of BIN1 in cardiac cells impairs delivery of calcium channels to the membrane and diminishes the intracellular calcium transient. According to this model, microtubules function as highways that carry newly synthesized calcium channels to BIN1-containing membrane. Once tethered to T-tubules by BIN1, the microtubules can deliver their calcium channel cargo. We postulate that this calcium channel delivery pathway is important to the regulation of cardiac calcium signaling and beat-to-beat cardiac function.

## Introduction

The BAR domain superfamily is composed of proteins involved in endocytosis, organelle biogenesis, cell division, and cell migration (review in [1]). As a member of the BAR domain superfamily, the tubulogenesis membrane scaffolding protein BIN1 (Amphiphysin 2) is known to induce membrane invagination [2],[3] and initiate tubulogenesis in skeletal muscle cells [4]. BIN1 deforms the membrane bilayer through interaction between its N-terminal positively charged BAR domain and acidic phospholipids within the cell membrane [4],[5]. Knowledge of the role of BIN1 in muscle cells includes evidence of BIN1 distribution on T-tubules of skeletal myocytes [4] and that constitutive knockdown of BIN1 in mice is perinatal lethal, with pathology revealing a hypertrophic dilated cardiomyopathy [6],[7]. However, despite these findings, little is known of the functional role of BIN1 in cardiomyocytes.

Since BIN1 knockdown results in cardiomyopathy [6], it is possible that BIN1 may play a role in regulating the cardiac calcium transient. During each heartbeat, calcium release from intracellular stores is achieved when trans-sarcolemmal calcium activates the ryanodine release channels on the sarcoplasmic reticulum (SR) [8]. The initial calcium influx occurs primarily through the L-type calcium channels with Cav1.2 as the pore-forming subunit. Trans-sarcolemmal calcium entry and activation of ryanodine receptors is a local phenomenon and, in cardiomyocytes, sarcolemmal Cav1.2 channels occur within 15 nm of their respective ryanodine receptors on the SR [9]. A major function of T-tubule invaginations of the sarcolemma, which are enriched with Cav1.2 channels [10],[11], is to bring the channels into close proximity of the ryanodine receptors, amplifying sarcolemmal calcium entry to a large calcium release from the SR. This process, which is known as calcium-induced calcium release (CICR) [12], is essential to each heartbeat and links electrical excitation of the myocyte and local calcium entry to its mechanical contraction. The mechanism for Cav1.2 localization to T-tubules remains unknown.

It is possible that locally enriched BIN1 may assist in the delivery of Cav1.2 channels in a manner similar to the role of adherens junctions in aided delivery of Connexin43 (Cx43) hemichannels to intercalated discs [13], a highly efficient trafficking pathway for polarized protein distribution. Therefore, depletion of BIN1 at T-tubule membrane after knockdown could result in mislocalization of Cav1.2, causing inefficient excitation-contraction (EC) coupling and lethal cardiomyopathy. Supporting evidence for this Cav1.2 localization hypothesis is that another BAR domain containing protein, endophilin, has been shown to complex with Cav1.2 at the plasma membrane [14]. Furthermore, BIN1 has been shown to interact not only with cortical actin [15],[16] but also with a microtubule plus end tracking protein [17]. These data indicate that BIN1 might be closely associated with growing microtubules, a key component of the trafficking machinery for targeted delivery.

In this study, we provide data supporting a role for BIN1 in tethering microtubules for direct delivery of L-type calcium channels to cardiac T-tubules. We observed that in both human and mouse cardiomyocytes, BIN1 and Cav1.2 colocalize at cardiac T-tubules (by fluorescence and electron microscopy immunogold labeling) and co-immunoprecipitate. Regarding delivery to T-tubules, we found that BIN1 tethers dynamic microtubules and forward trafficking of Cav1.2 channels is microtubule dependent. In reductionist atrial myocyte and non-myocyte cell systems, BIN1 is sufficient to form membrane invaginations and distribute Cav1.2 to these BIN1-containing membrane regions (visualized by total internal reflection microscopy, TIRFm). The delivery results in non-myocyte cells suggest that Cav1.2 delivery to BIN1 is independent of other myocyte-specific organelles and proteins. To rule out that the sarcolemmal invaginations themselves and not BIN1 are sufficient for Cav1.2 delivery, we created C-terminal truncated BIN1, which fails to attract Cav1.2 yet still inducing membrane invagination. In isolated primary mouse ventricular cardiomyocytes, disruption of this delivery mechanism by BIN1 knockdown results in less surface expression of Cav1.2 and abnormal calcium transient development.

Our findings indicate that the membrane curvature protein BIN1 can form membrane invaginations and is localized to cardiac T-tubules, providing an anchor for microtubules that allows targeted delivery of Cav1.2 channels and regulation of the cardiac calcium transient. A role of BIN1 in facilitating microtubule-based antegrade delivery of membrane protein traffic adds an important facet to the multifunctional BAR domain family. Furthermore, our findings suggest that microtubule-based delivery of Cav1.2 to BIN1 is significant to cardiac calcium regulation.

## Results

### BIN1 Is Distributed along Cardiac T-Tubules and Colocalizes with Cav1.2

**Figure 1:**
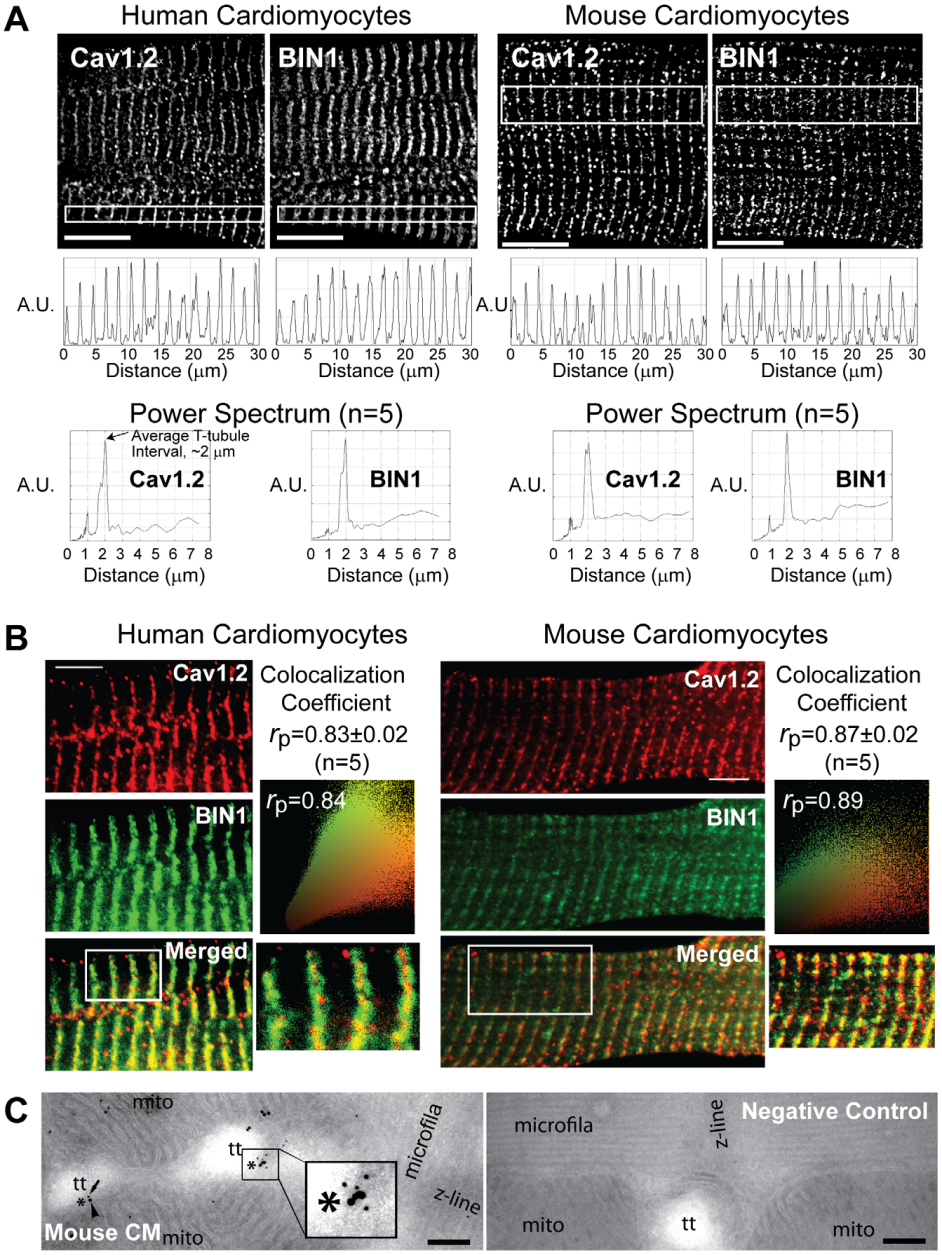
BIN1 colocalizes with Cav1.2 at T-tubules in cardiomyocytes. (A) Confocal image (100×) of human (left) and mouse (right) adult cardiomyocytes. The cells were fixed and stained with mouse anti-BIN1 or rabbit anti-Cav1.2. Two-dimensional frames of Cav1.2 and BIN1 are shown in the top panel. Cardiomyocyte fluorescence intensity profiles along the cardiomyocyte longitudinal axis are presented in the middle panel. The bottom panel is the power spectrum over spatial distance averaged from five cardiomyocytes, which indicate that both BIN1 and Cav1.2 signals occurs at every 2 µm (fundamental peak occurs at ∼2 µm). Note the small peak at 1 µm is a harmonic of the fundamental peak at 2 µm (scale bar: 10 µm). (B) Confocal images (100×) of human (left) and mouse (right) cardiomyocytes stained with mouse anti-BIN1 (green) and rabbit anti-Cav1.2 (red) reveal colocalization between BIN1 and Cav1.2 along T-tubules (scale bar: 5 µm). Pearson colocalization coefficient and scatter plot between BIN1 and Cav1.2 are also shown in this panel. (C) Electron microscopy image of adult mouse cardiomyocytes fixed and immunogold labeled for BIN1 (small dots) and Cav1.2 (large dots) (scale bar: 200 nm) (left). As seen in the enlarged image, BIN1 and Cav1.2 occurs within 50 nm on T-tubule membranes. The negative control image without primary antibodies incubation is shown at the right panel.

To understand the cellular distribution of BIN1 in mammalian cardiomyocytes, we dissociated non-failing human cardiomyocytes from freshly explanted human hearts with normal left ventricular function, as well as normal adult mouse cardiomyocytes. After fluorescence immunostaining, the cardiomyocytes were imaged at Z-depth increments of 0.1 µm with a spinning disc confocal microscope and viewed in two-dimensional frame views along the longitudinal axis. BIN1 has a nuclear localization, as previously reported, in embryonic hearts (Figure S1) [6], but elsewhere in the cardiomyocyte, a typical T-tubule distribution pattern of BIN1 emerges that is similar to Cav1.2 distribution (Figure 1A, first row). Representative fluorescence intensity profiles along the longitudinal axis of cardiomyocytes are in the second row of Figure 1A. Note that there is a fluorescence signal peak approximately every 2 µm, which corresponds to the T-tubule distribution of the protein. Power spectrum analysis [18] confirms that the fundamental periodicity of Cav1.2 is 2 µm (Figure 1A, third row), which is consistent with previously reported cardiac T-tubule intervals [18],[19]. BIN1 shows the same spatial periodicity as Cav1.2 in human and mouse (Figure 1A) cardiomyocytes. Cx43, which localizes at intercalated discs at the longitudinal ends of cardiomyocytes, does not have the same spatial periodicity and serves as a negative control (Figure S2). The data of Figure 1A indicate that BIN1 is localized along T-tubules in cardiomyocytes.

Next, we quantified colocalization between Cav1.2 and BIN1. In Figure 1B, immunolabeling of BIN1 (green) and Cav1.2 (red) is shown in subsections of both human and mouse cardiomyocytes. Full cardiomyocyte views of colocalization between BIN1 and Cav1.2 are shown in Figure S3. The data indicate that BIN1 significantly colocalizes with Cav1.2, primarily at T-tubules. For negative control studies, Cav1.2 does not have significant colocalization with Cx43 (Figure S4). To confirm spatial coincidence, we used transmission electron microscopy with dual immunogold labeling to identify Cav1.2 and BIN1 on T-tubule ultrastructures in adult mouse cardiomyocytes. Results in Figure 1C (left panel) indicate that BIN1 (small 10 nm dots) and Cav1.2 (large 15 nm dots) are enriched and occur within 10–50 nm of each other at T-tubular membrane structures.

### BIN1 Tethers Dynamic Microtubules Involved in Antegrade Trafficking of Cav1.2

**Figure 2 pbio-1000312-g002:**
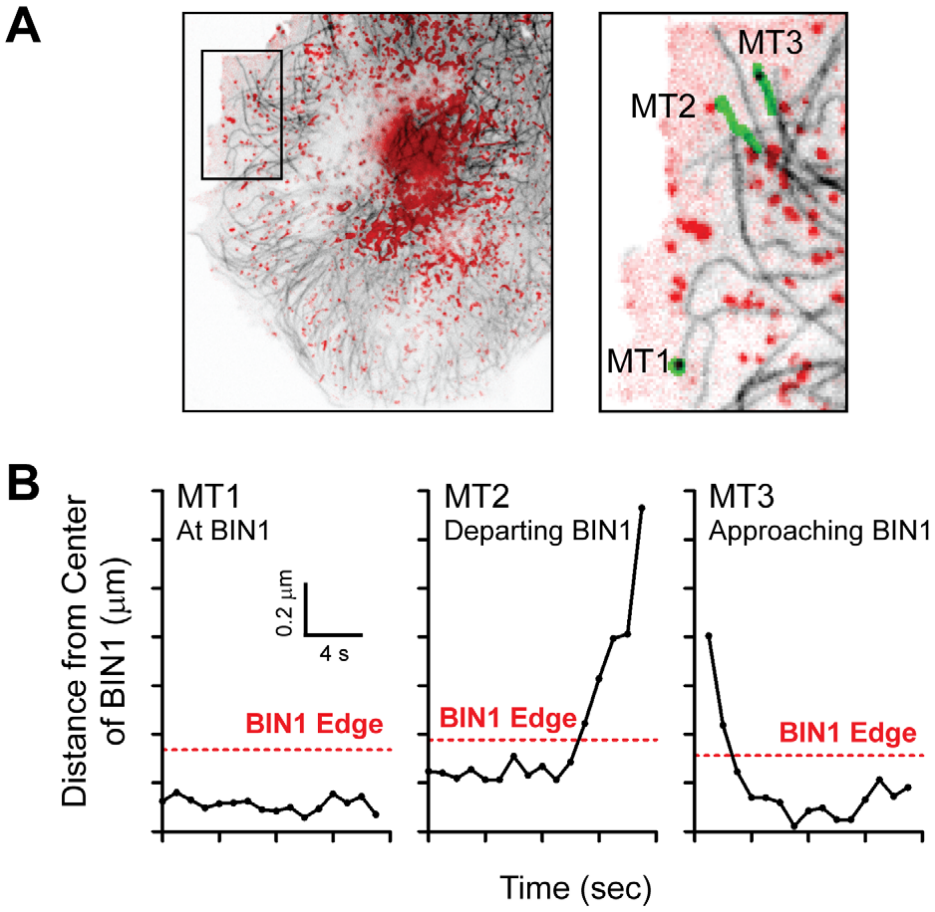
BIN1 tethers dynamic microtubules. (A) HeLa cells were transfected with α-Tubulin-GFP and BIN1-mCherry. The overlay pictures of BIN1 (red) and microtubules (black) are shown in the left panel. The right image is an enlarged subsection of the left image. Three microtubule travel paths (MT1, MT2, and MT3) are also highlighted in green in the subsection. (B) Graphs of each microtubule travel path. BIN1 edge (within 0.2 µm of BIN1 structure) is highlighted with a red dotted line in each graph.

The data in Figure 1C indicate close approximation of Cav1.2 and BIN1 in isolated cardiomyocytes but do not reveal how the proteins achieve such localization. There is significant support for membrane ion channel delivery occurring via microtubules [13],[20],[21]. To address whether BIN1 serves as a microtubule anchoring site to allow Cav1.2 delivery, we first studied microtubule behavior in the vicinity of BIN1 in HeLa cells, which are permissive to high-resolution imaging. HeLa cells were transfected with α-tubulin-GFP and BIN1-mCherry. Introduction of exogenous BIN1 forms membrane invaginations as previously reported in other non-myocyte cell types [4]. Twenty-four hours post-transfection, microtubule dynamics were recorded with spinning disc confocal microscopy for 2 min with a frame rate of 1 s. As seen in the enlarged panel of the overlay between BIN1 (red) and microtubules (black lines) in Figure 2A, microtubules tether at BIN1 structures. Three representative microtubule travel paths involving a microtubule that remains at BIN1 (MT1), a microtubule that departs BIN1 (MT2), and a microtubule that approaches BIN1 (MT3) are also indicated in green. For each of these three microtubules, the distance between the microtubule tip and the center of the closest BIN1 structure is plotted over time in Figure 2B. In each graph, the distance within 0.2 µm of the respective BIN1 structure is highlighted in red dotted lines. MT1 has paused at BIN1 structure for the whole 2 min imaging window and has relatively little movement. However, MT2 pauses and hovers at BIN1 and, upon leaving, picks up velocity, while MT3 approaches BIN1 with high velocity before it slows down as it comes into contact with BIN1. The dynamic movements of MT1, MT2, and MT3 are shown in Video S1. In addition, the overall microtubule dynamics tabulated from 15 microtubules of four individual cells are presented in Table 1. These data indicate that overall tip velocity is 5× faster (0.15 µm/s versus 0.03 µm/s) when the microtubules are not in the proximity of BIN1. This increased overall velocity consists of not only faster growth and shortening velocities but also less frequent pauses. The data from Figure 2 and Table 1 suggest that microtubules are tethered by BIN1 structures.

**Table 1:** Microtubule dynamics in the proximity of BIN1 structures.

	At BIN1 (*n* = 20)	Away from BIN1 (*n* = 19)	*p* Value
Overall velocity (µm/s)	0.03 ± 0.01	0.15 ± 0.02	4E-06
Growth velocity (µm/s)	0.13 ± 0.01	0.21 ± 0.02	6E-04
Pause fraction	0.20	0.11	0.004
Shortening velocity (µm/s)	0.14 ± 0.01	0.23 ± 0.04	0.003

Table 1 contains the overall travel velocity, growth, and shortening velocities, as well as pausing events of microtubules whose tips are within 0.2 microns of, or away from, BIN1 structures. *n* is number of events. Data are from 15 microtubules in four different cells.

**Figure 3:**
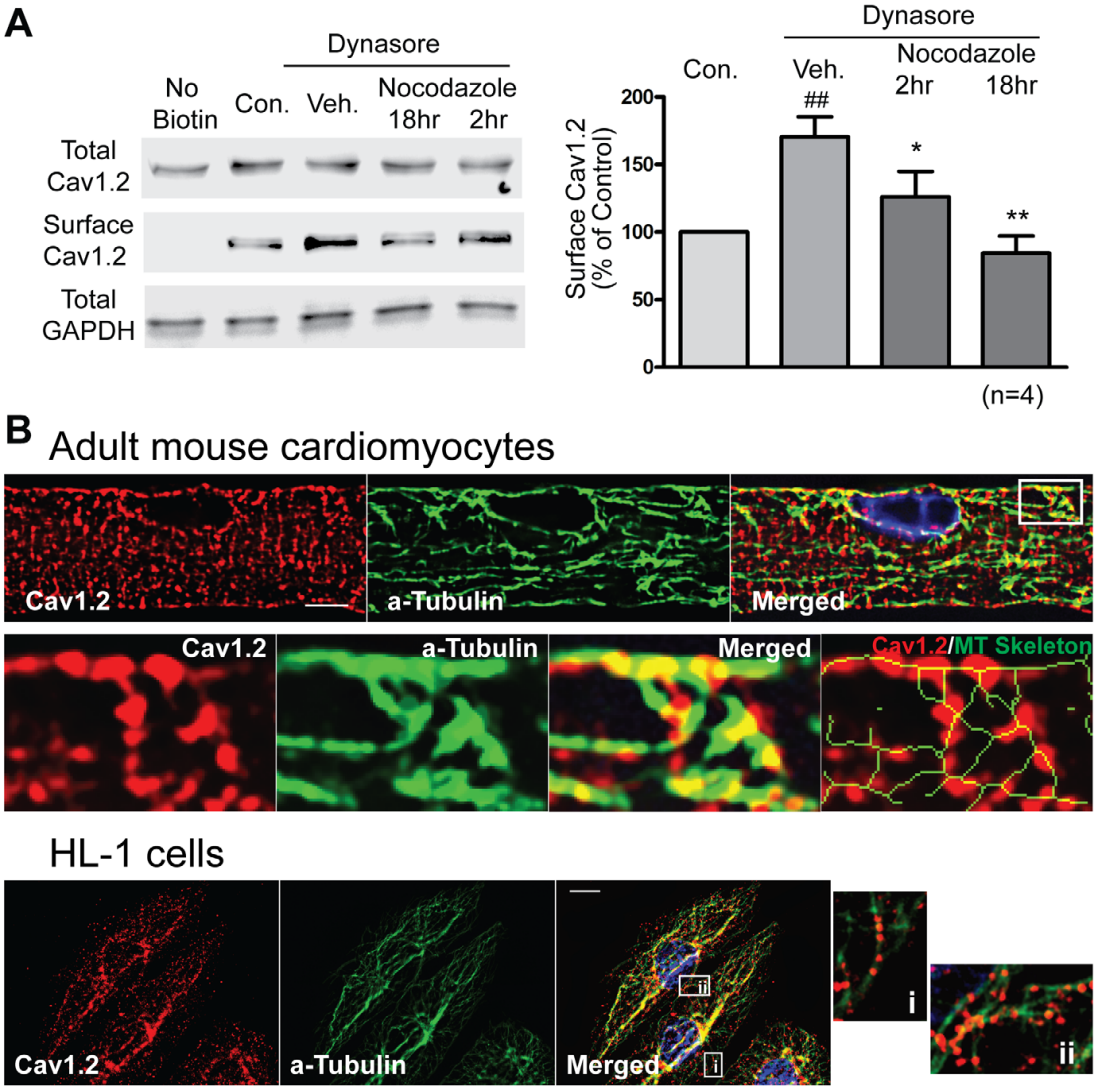
Antegrade trafficking of Cav1.2 is microtubule dependent. (A) Surface biotinylation of adult mouse cardiomyocytes indicates that nocodazole (30 µM) progressively reduces surface Cav1.2 expression in the presence of an endocytosis inhibitor dynasore (20 µM). Note that dynasore alone significantly increases surface expression of Cav1.2 by blocking dynamin-dependent endocytosis of Cav1.2 in cardiomyocytes. (B) Top panel: Confocal images (100×) of mouse cardiomyocytes stained with rabbit anti-Cav1.2 (red) and mouse anti-α-tubulin (green) reveal localization of Cav1.2 on microtubule network (scale bar: 5 µm). Bottom panel: Deconvolution of wide-field image of HL-1 cells stained with Cav1.2 (red) and α-tubulin (green). Merged image shows localization of Cav1.2 to the microtubule network. Enlarged pictures (right) indicate that Cav1.2 is distributed along microtubules (## *p*<0.01 when compared to control group, * *p*<0.05, ** *p*<0.01, when compared to vehicle group, Student's *t* test).

To evaluate if microtubules are involved in antegrade trafficking of Cav1.2 channels, we exposed live primary adult ventricular cardiomyocytes to the microtubule disruptor nocodazole in the presence of dynasore, a specific dynamin GTPase inhibitor that blocks endocytosis [22]. Expression of surface membrane-bound Cav1.2 was assayed by cell surface biotinylation (Figure 3A). Dynasore treatment alone increases surface expression of Cav1.2, indicating inhibition of Cav1.2 endocytosis. In the presence of both dynasore and nocodazole, Cav1.2 surface expression progressively decreases, further suggesting that microtubule disruption reduces forward trafficking of Cav1.2 to the plasma membrane. To confirm that delivery of Cav1.2 to T-tubules is microtubule dependent, the cellular distribution of Cav1.2 in cardiomyocytes subjected to nocodazole was studied by immunoconfocal microscopy. As seen in Figure S5, nocodazole decreases Cav1.2 surface expression not only at T-tubules (Figure S5, bottom right) but also at global sarcolemma containing non-T-tubule membrane (Figure S5, bottom left). The total cellular protein expression level of Cav1.2 is not changed by nocodazole (Western blot in Figure S5, top panel). Microtubule-dependent trafficking of Cav1.2 is further supported by the localization of Cav1.2 vesicles along the microtubule network in the vicinity of a T-tubule in adult mouse cardiomyocytes (Figure 3B, top panel). To better visualize microtubules and Cav1.2, we used the cardiomyocyte-derived HL-1 cell line that has a morphology amenable to high-resolution imaging [23] and find that Cav1.2 distributes along the microtubule network (Figure 3B, bottom panel). Comparable biotinylation results confirm microtubule-dependent surface expression of Cav1.2 in HL-1 cells (Figure S6).

From the data in Figures 1–3, it appears that BIN1 is enriched along cardiac T-tubules and closely associated with Cav1.2. Furthermore, BIN1 tethers to plasma membrane dynamic microtubules, which deliver Cav1.2 to the plasma membrane. Therefore, it is possible that BIN1 is a T-tubule anchor site for targeted delivery of Cav1.2 through the interaction between BIN1 and growing microtubules.

### Cav1.2 Concentrates at BIN1-Induced Membrane Invaginations

**Figure 4:**
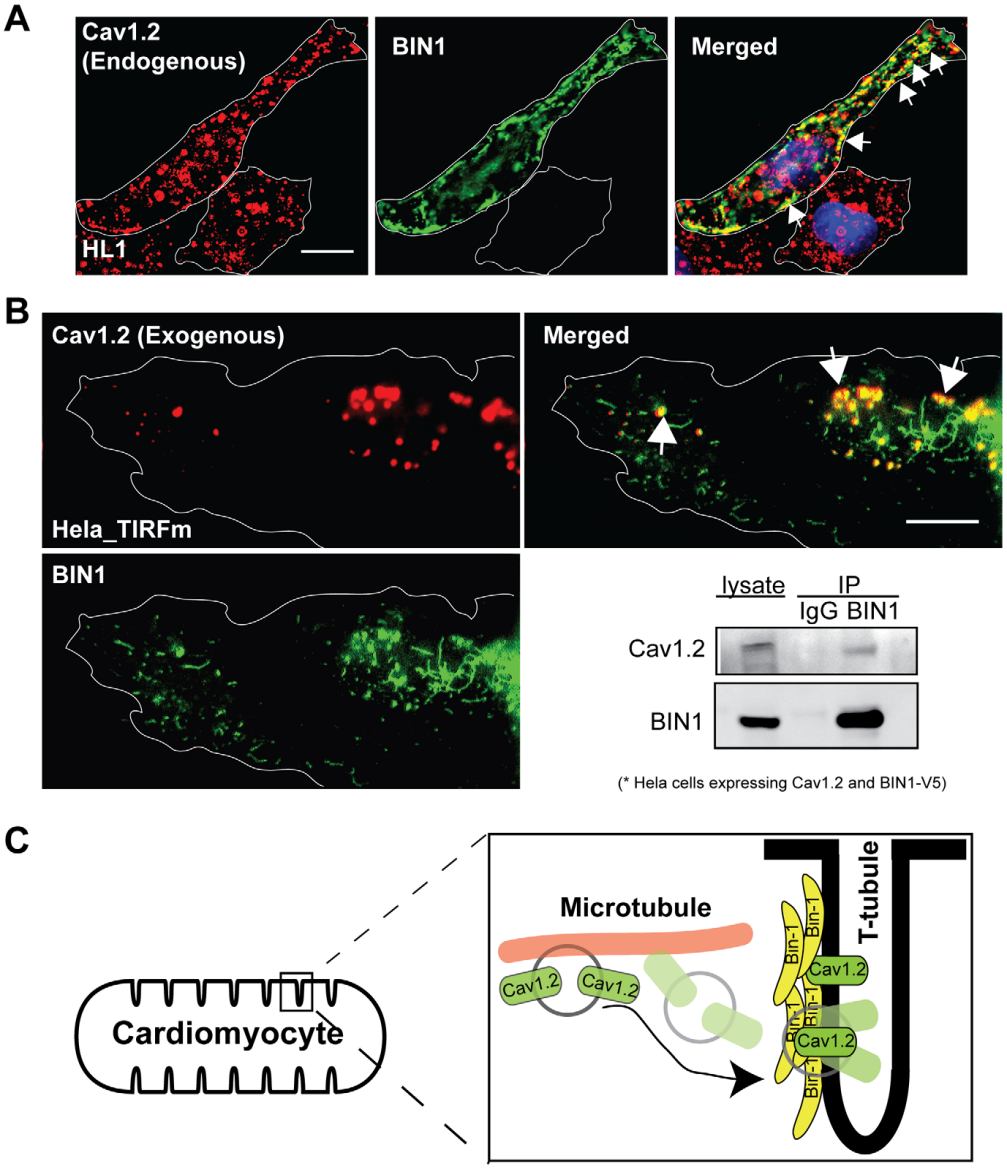
Cav1.2 is targeted to BIN1-induced membrane structures. (A) Deconvolution of wide-field image (100×) of BIN1 transfected HL-1 cells indicates endogenous Cav1.2 (red) colocalizes with exogenous BIN1 (green) (scale bar: 5 µm). (B) TIRFm images of a HeLa cell transfected with Cav1.2-GFP (red) and BIN1-mCherry (green) reveal colocalization between BIN1 and Cav1.2 at the cell periphery (scale bar: 5 µm). This panel also includes co-immunoprecipitation between overexpressed BIN1-V5 (IP) and Cav1.2 (IB) in HeLa cells. (C) A schematic of dynamic microtubules delivering Cav1.2 to BIN1 at T-tubules.

To test the exclusivity of the relationship between Cav1.2 and BIN1, we explored whether Cav1.2 could be targeted to exogenous BIN1-induced membrane invaginations in cell lines lacking a developed T-tubule system. HL-1 cells are myocytes that express endogenous Cav1.2 but do not have a developed T-tubule system. Introduction of exogenous BIN1 generates membrane invaginations of cell membrane that appear as linear streaks [4], as seen in Figure 4A (green, with Cav1.2 in red). As indicated by the structures near the arrows in the right panel of Figure 4A, Cav1.2 localizes to exogenous BIN1, just as Cav1.2 localizes to endogenous BIN1 in primary cardiomyocytes seen in Figure 1. To confirm that membrane delivery of Cav1.2 to BIN1 can occur in non-myocyte cells, we evaluated surface expression patterns of exogenous Cav1.2 in HeLa cells expressing exogenous BIN1. In order to resolve BIN1 structures at the level of plasma membrane, we used TIRFm, which limits the imaging depth to within 50–100 nm. Using Cav1.2 and BIN1 tagged with spectrally distinct fluorophores, we performed a brief time lapse capture, with representative results shown in Figure 4B. The data indicate that BIN1-induced structures (green) attract surface Cav1.2 (red), causing local enrichment of calcium channel. Thereby, in the absence of other myocyte structures as well as the absence of endogenous Cav1.2, ectopic expression of BIN1 is sufficient to concentrate surface Cav1.2. The possibility of close biochemical association between BIN1 and Cav1.2 in HeLa cells is further supported by co-immunoprecipitation of V5-tagged BIN1 and Cav1.2 (Figure 4B). In summary, we find that microtubule-based delivery of Cav1.2 to tubular membrane invaginations is BIN1 dependent and is independent of other myocyte-specific structures and proteins (Figure 4C).

### Cav1.2 Targeting Requires BIN1, Not Membrane Invaginations

**Figure 5:**
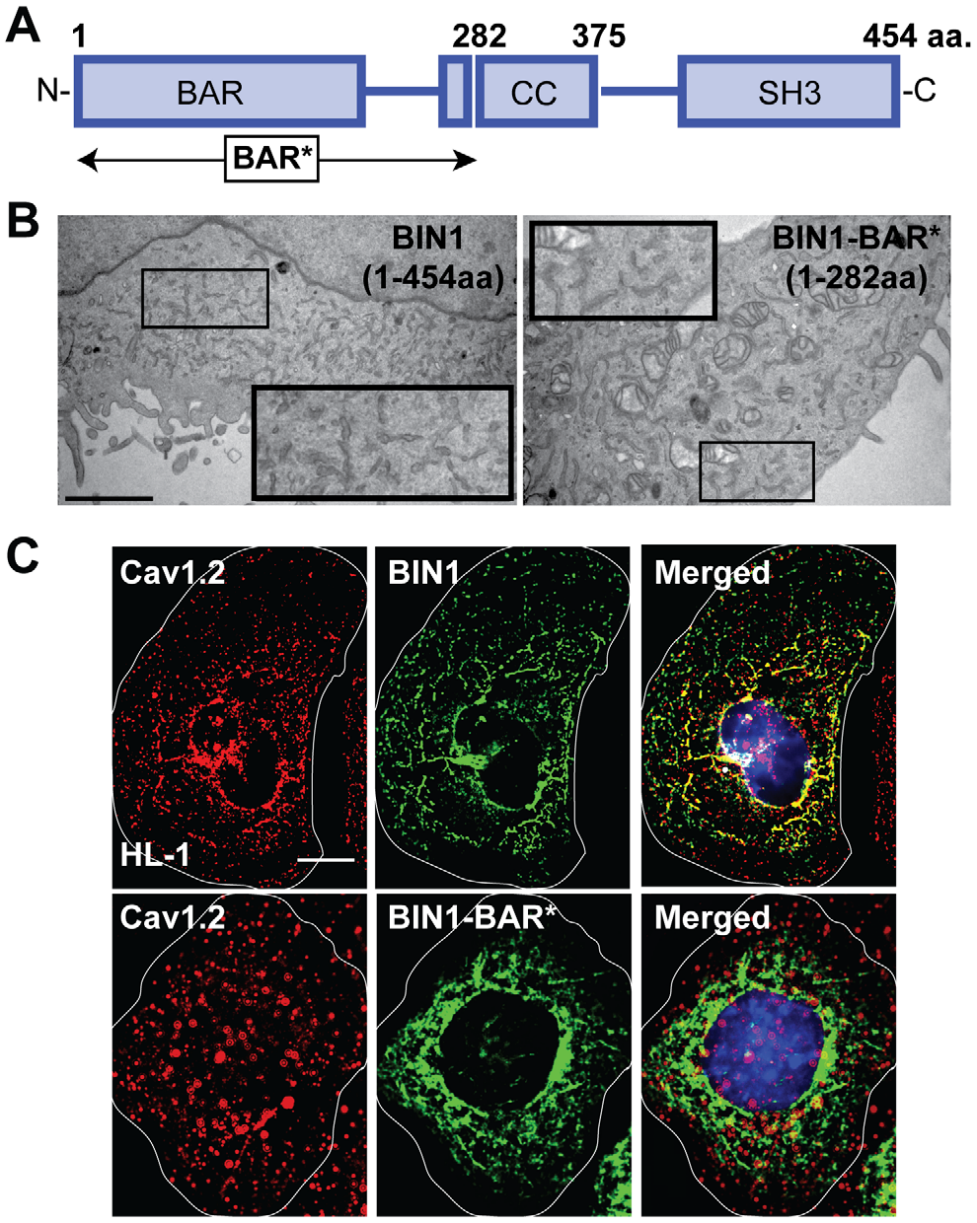
Cav1.2 is targeted to BIN1, not membrane invaginations. (A) Domain map of wild-type BIN1 (BIN1). BIN1-BAR* (1-282 aa) contains the BAR domain and the sequence upstream of the coiled-coil region that is necessary for inducing membrane invagination. (B) Electron microscopy images indicate that BIN1 and BIN1-BAR* form similar membrane invaginations (dark linear tubules). (C) Deconvolved wide-field image of HL-1 cells transfected with BIN1 or BIN1-BAR*(1-282 aa). Co-staining between endogenous Cav1.2 (red) with transfected exogenous BIN1 or BIN1-BAR* (green) indicates that Cav1.2 localizes to BIN1 structures but not BIN1-BAR* structures (scale bar: 5 µm).

**Figure 6:**
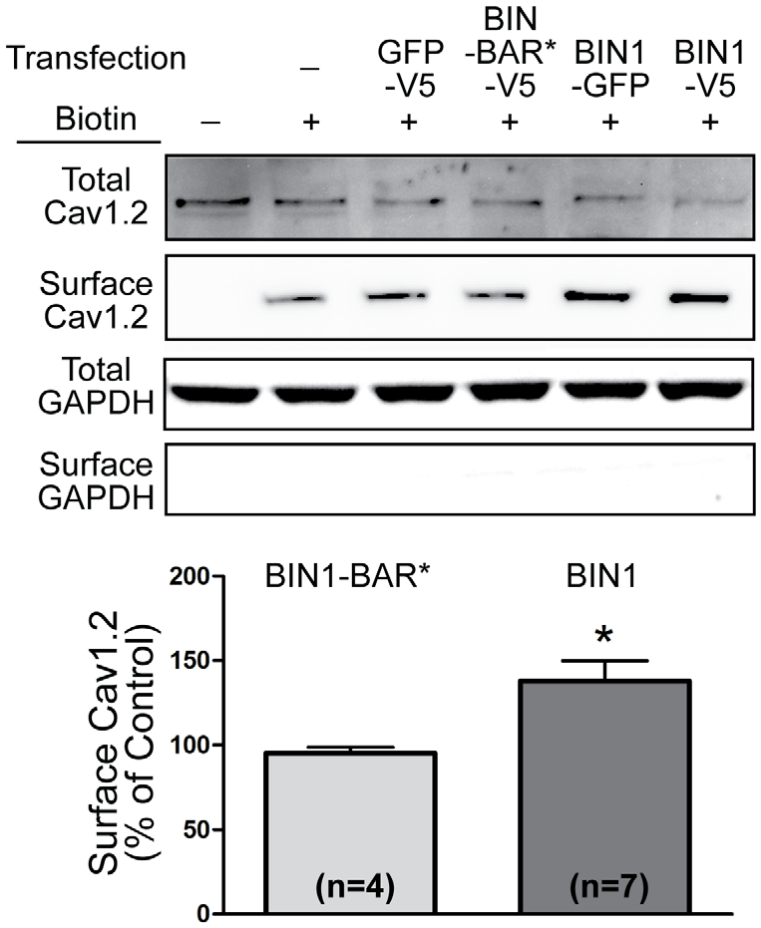
Full-length BIN1 causes Cav1.2 surface expression. Surface biotinylation of Cav1.2 in HL-1 cells transfected with either BIN1 or BIN1-BAR* reveals that full-length BIN1 is required to cause surface expression of Cav1.2 (* *p*<0.05, Student's *t* test).

To confirm that it is specifically BIN1, and not the BIN1-induced membrane invaginations, that localizes Cav1.2, we used a truncation mutant of BIN1. Full-length BIN1 (1-454 aa) has an N-terminal BAR domain followed by a coiled-coil linkage domain and a C-terminal SH3 domain (Figure 5A) [4],[24]. Following precedent [4], we created a C-terminal truncated BIN1-BAR* (1-282 aa), which retains the ability to induce membrane invagination (the electron microscopy membrane structures are shown in Figure 5B). However, BIN1-BAR* loses the ability to attract endogenous Cav1.2 to the nascent membrane invaginations such as those in HL-1 cells (Figure 5C). With full-length BIN1 (top row), endogenous Cav1.2 is distributed along BIN1 structures. In contrast, Cav1.2 (red) has poor colocalization with BIN1 structures (green) in cells transfected with BIN1-BAR* (bottom panel). The effect of full-length BIN1 and BIN1-BAR* on Cav1.2 surface targeting was further tested by a biochemical surface biotinylation assay. As in Figure 6, unlike BIN1-BAR*, full-length BIN1 has greater surface expression of Cav1.2. Thus, targeting of Cav1.2 to membrane invaginations requires full-length BIN1. It appears that BIN1 recruitment of Cav1.2 involves a domain distinct from that which induces membrane curvature. To determine specificity of BIN1 to Cav1.2, we repeated the surface biotinylation assay for the sodium calcium exchanger 1 (NCX1), which is also a T-tubule localized channel. As seen in Figure S7, BIN1 fails to increase surface expression of NCX1, indicating that BIN1-based delivery has specificity for Cav1.2.

### In Mouse Cardiomyocytes, BIN1 Knockdown Delays Calcium Transient

**Figure 7:**
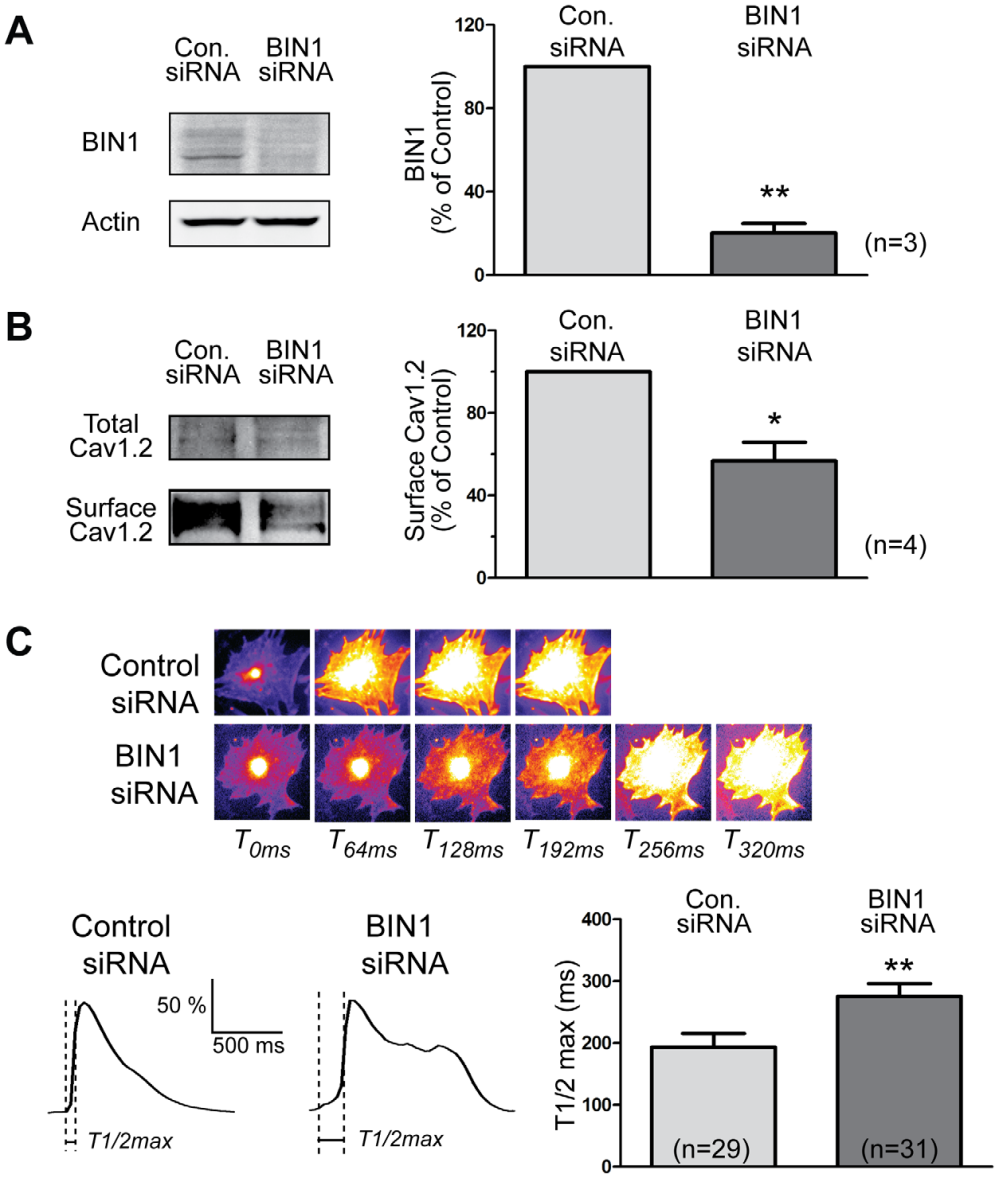
BIN1 knockdown delays calcium transient development in mouse cardiomyocytes. (A) Western blot indicates an 80% knockdown of BIN1 protein by siRNA in differentiated mouse cardiomyocytes. (B) Surface biotinylation of Cav1.2 in these primary cardiomyocytes indicates a 45% reduction of surface Cav1.2 after BIN1 knockdown. (C) Live cell calcium imaging in differentiated cardiomyocytes indicates that BIN1 knockdown also delays calcium transient development in these cells. Average time to 50% maximal fluorescence intensity (T1/2 max) of calcium transient is presented in the left panel (* *p*<0.05, ** *p*<0.01, Student's *t* test).

We then investigated whether disruption of such a T-tubule-targeting mechanism of Cav1.2 impacts cardiomyocyte function. Although T-tubules are only partially developed in freshly dissociated neonatal cardiomyocytes [25],[26], earlier studies by electron microscopy show T-tubules develop after 3 days differentiation in culture [27] along with redistribution of Z-line-associated cytoskeleton proteins for Z-line organization [28]. Recent studies also find that in cultured differentiated neonatal cardiomyocytes, the dihydropyridine receptor [29] and other components of the calcium-release and uptake machinery [30], as well as other T-tubule proteins [31], develop a typical T-tubule staining pattern. Similarly, we observed T-tubule-type staining in cells dissociated at postnatal day three or four and allowed to differentiate in culture for a week. These structures were enriched with both Cav1.2 and BIN1 (Figure S8A). Furthermore, BIN1 mRNA expression in postnatal mouse heart tissue is similar to that in adult heart (Figure S8B). Using this differentiated mouse cardiomyocyte population, BIN1 siRNA successfully decreases BIN1 expression by 80%, as assayed by Western blot in Figure 7A. As a result of BIN1 knockdown, surface Cav1.2 is reduced by 45%, although the total cellular protein expression of Cav1.2 remains similar (Figure 7B). To assay the effect on cardiomyocyte calcium transients, we loaded the cells with a fluo 4-AM and imaged with a wide-field epifluorescence microscope. As seen in Figure 7C, loss of BIN1 results in a significant slowing of calcium transient development, indicating reduced CICR. The slowing of calcium transient development is quantified by measuring the time to reach 50% of peak calcium concentration (T1/2 max). BIN1 knockdown delayed T1/2 max by 40% (bar graph, Figure 7C). The data in Figure 7 indicate that knockdown of BIN1 reduces the surface expression of Cav1.2, impairing the intracellular cardiac transient, and that BIN1 is necessary to maintain normal calcium signaling in the heart.

## Discussion

The primary finding of this study is the identification of a novel role for BIN1 as a T-tubule anchoring protein accepting antegrade delivery of Cav1.2. Immunocytochemical staining indicates that BIN1 colocalizes with Cav1.2 along T-tubules in primary adult human and mouse cardiomyocytes (Figure 1). Dual immunogold transmission electron microscopy images reveal that Cav1.2 and BIN1, which co-immunoprecipitate (Figure 4), cluster together within ∼10–50 nm on T-tubules (Figure 1C). Regarding delivery of Cav1.2 to T-tubules, there is significant support for membrane ion channel delivery occurring via microtubules [13],[20],[21]. In exploring the forward trafficking mechanism of Cav1.2, we find that microtubules are required for the delivery of Cav1.2 (Figure 3) and that BIN1 anchors microtubules (Figure 2, Table 1), which can provide offloading of Cav1.2-containing vesicles to T-tubule membrane.

BIN1 is a member of the BAR domain containing protein family, which has a role in membrane bilayer deformation at endocytic sites through interaction between their N-terminal positively charged BAR domains and acidic phospholipids within cell membrane [5]. Fluorescence and electron microscopy reveal that a human BIN1 construct can induce enormous membrane invaginations in both non-T-tubule-forming atrial HL-1 cells and non-cardiac HeLa cells (Figures 4A, 4B, 5B), as previously reported in other cell types [4]. If BIN1 at cardiac T-tubules is closely associated with Cav1.2 (Figure 1) and dynamic microtubules (Figure 2), it is possible that BIN1 alone is sufficient to target microtubule-transported Cav1.2. In myocyte HL-1 cells, overexpression of exogenous BIN1 changes the cellular distribution of endogenous Cav1.2 and relocalizes them to nascent BIN1-induced membrane invaginations (Figure 4A). Furthermore, loss of BIN1 in cardiomyocytes reduces surface expression of Cav1.2 (Figure 7B). Such delivery is not myocyte dependent. In HeLa cells, which are devoid of the cardiac-specific cellular ultrastructures and machinery, overexpression of BIN1 is sufficient to localize exogenous Cav1.2 to the cell periphery on BIN1 membrane structures as resolved by simultaneous dual-color TIRFm (Figure 4B). Co-immunoprecipitation of BIN1 and Cav1.2 (Figure 4B) further indicates that they are present in the same protein complex. This physical association between BIN1 and Cav1.2 further supports the model that BIN1 serves as a membrane anchor site for Cav1.2 (Figure 4C).

T-tubules are a well-organized membrane structure in which it is unknown how the T-tubule-related proteins localize there, specifically for Cav1.2. How could we then exclude the possibility that the membrane invaginations alone are sufficient to cause Cav1.2 delivery to T-tubules independent of BIN1? As previously established [4], the extended BAR domain (BAR*, amino acid 1–282, Figure 5A) of BIN1 is sufficient for inducing membrane invagination (Figure 5B), despite the absence of other domains responsible for protein-protein interaction. Distinct from full-length BIN1, BIN1-BAR* neither redistributes Cav1.2 (Figure 5C) nor causes Cav1.2 surface expression (Figure 6). It appears that BIN1 recruitment of Cav1.2 involves a domain distinct from the one that induces membrane curvature, as suggested by endophilin binding to Cav1.2 in its non-BAR coiled-coil region [14].

The BAR domain superfamily has previously been associated with anchoring cortical actin at the plasma membrane [15],[16]. This study introduces microtubule anchoring as well (Figure 2). There may be a general role for BAR domain proteins in allowing antegrade trafficking and localization of membrane-bound proteins. Furthermore, the mechanism of targeting Cav1.2 to T-tubules may be responsible for diseases associated with genetic BIN1 dysfunction. In mice, BIN1 knockout causes perinatal lethal cardiomyopathy [6], and a mutation in the same 2q14-22 locus of BIN1 is associated with familial cardiomyopathy in humans [32]. Loss of function mutations in BIN1 also results in centronuclear peripheral myopathy [24] characterized by muscle weakness, which could be explained by calcium dysregulation. Future studies will be required to investigate whether calcium channel trafficking and localization are altered in these diseases.

With regard to cardiac myocytes, our findings constitute a new understanding of calcium channel regulation. In order to allow trans-sarcolemmal calcium to reach the intracellular ryanodine receptors, Cav1.2 channels must be localized at T-tubules [9]. It has been estimated that T-tubule calcium channels contribute 80%–90% of the total cellular calcium current [10],[11]. Furthermore, Cav1.2 experiences a high turnover, with pulse chase experiments indicating a half-life as short as 3.5 h [33]. The need for specific localization with a rapid turnover implicates that channel delivery is an important and highly regulated aspect of Cav1.2 channel function. Indeed, our data indicate that T-tubule targeting of Cav1.2 by BIN1 is critical in calcium handling and regulation in cardiomyocytes. As seen in Figure 7, loss of BIN1 reduces surface Cav1.2 and delays calcium transient development in primary cardiomyocytes. The data indicate that BIN1 functions as a T-tubule-membrane-anchoring site for microtubules to deliver Cav1.2, thereby ensuring proper control of cardiac EC coupling.

Moreover, the mechanistic understanding of Cav1.2 trafficking to T-tubules by our current study not only provides insight into calcium regulation in normal hearts but also has significant implications in the pathogenesis of diseases with altered calcium dynamics such as congestive heart failure (CHF). In failing heart, the intracellular calcium transient of ventricular cardiomyocytes has a low amplitude and slow decline [34]–[36], resulting in compromised contraction [37]. Multiple factors downstream of calcium entry through Cav1.2 have been identified in failing muscle that contribute to changes in the calcium transient, including dysfunction in calcium removal [38],[39] and, more recently, phosphorylation and perturbation of the ryanodine release channels [40],[41]. There have also been reports that dyssynchronous CICR may exist in failing cardiomyocytes and contribute to defective EC-coupling gain in failing heart [42],[43]. Since localization of L-type calcium channels is critical for synchronous CICR, loss or mislocalization of these channels in the local microenvironment might lead to defective CICR and abnormal heart function. In fact, human CHF has reduced L-type calcium channel density in the sarcolemma [44], and a canine model of heart failure is associated with remodeling of both Cav1.2 distribution and T-tubule structure [45]. In this study, we found that BIN1-based microtubule targeting affects Cav1.2 localization and intracellular calcium dynamics (Figure 7). It will be interesting in future studies to explore the role of BIN1 regulation in heart failure.

## Materials and Methods

### Plasmids, Cell Culture, and Transfection

Human BIN1 (Isotype 8) cDNA was obtained from Origene. Full-length BIN1-8(1-454 aa) and BIN1-BAR*(1-282 aa) were then amplified and cloned into pDONR/Zeo (Invitrogen) using Gateway BP cloning to generate entry clones. The genes were subsequently inserted into pDest-eGFP-N1, pDest-mCherry-N1 (converted vectors originally from Clontech), and pcDNA3.2-V5-Dest by Gateway LR cloning. Human Cav1.2 was obtained from Origene. Human β2b and rabbit α2δ1 were generously provided by Dr. Michael Sangunetti. N-terminal GFP-Cav1.2 was generously provided by Dr. Kurt Beam, and C-terminal Cav1.2-GFP was described previously [46]. Non-targeting and BIN1-specific siRNA were obtained from Dharmacon.

HeLa cells and mouse atrial HL-1 cells were cultured in DMEM and Claycomb medium under standard mammalian cell conditions. FuGene 6 (Roche) was used for cDNA transfections in HeLa cells. Lipofectamine (Invitrogen) was used for cDNA transfections in HL-1 cells.

### Immunostaining

Dissociated cardiomyocytes were allowed to attach to laminin-precoated glass coverslips before fixation. For all immunocytochemistry, cells were fixed in methanol at −20°C for 5 min. For immunohistochemistry, cryosections were fixed in ice-cold acetone for 10 min. After fixation, cardiomyocytes were permeablized and blocked with 0.5% Triton X-100 (Sigma) and 5% NGS in PBS for 1 h at room temperature. For BIN1 and Cav1.2 staining, the cells were incubated with mouse anti-BIN1 (1∶50, Sigma) and rabbit anti-Cav1.2 (1∶50, Alomone) overnight at 4°C. Similar protocol without permeablization was used for BIN1 and Cav1.2 staining in myocardium cryosections. For co-staining of Cav1.2 and α-tubulin in HL-1 cells, after fixation, the cells were permeablized with 0.1% Triton X-100 for 15 min and blocked with 5% NGS for 1 h. The cells were then incubated with rabbit anti-Cav1.2 (1∶50, Alomone) overnight at 4°C followed by mouse monoclonal to α-Tubulin (1∶500, Sigma) for 1 h at room temperature. After several washes with PBS post-primary antibody incubation, cells were then incubated with goat anti-mouse and -rabbit IgG conjugated to AlexaFluor 488 and 555, respectively. Cells were then fixed and mounted with DAPI containing ProLong gold.

### Wide-Field Epifluorescence, TIRF, and Spinning Disc Confocal Microscopy

All imaging was performed on a Nikon Eclipse T*i* microscope with a 100× 1.49 NA TIRF objective and NIS Elements software. Deconvolution of images was performed using Autoquant software (Media Cybernetics). High-resolution cardiomyocyte images were obtained by a spinning disc confocal unit (Yokogawa CSU10) with DPSS lasers (486, 561) generated from laser merge module 5 (Spectral applied research, CA) and captured by a high-resolution Cool SNAP HQ^2^ camera (Photometrics). Multiple wavelength TIRF was achieved with Dual-View emission splitter (Optical Insights). High-sensitive Cascade II 512 camera (Photometrics) was used for TIRF image capture.

For BIN1 and Cav1.2 distribution, isolated human and mouse cardiomyocytes were imaged at Z-depth increments of 0.1 µm and reconstructed to generate three-dimensional volume views and frame view along the longitudinal axis using NIS Element Software. To access Cav1.2 and BIN1 colocalization by TIRFm, HeLa cells were plated overnight and co-transfected with pDest-BIN1-mCherry, and Cav1.2-GFP along with β2b and a2δ1. Dual channel TIRF time lapse sequences of 1 min were acquired at an exposure of 200 ms per image at a rate of 1 frame per second. After acquisition, the total 61 frames were z-projected into one frame using ImageJ (NIH).

For live-cell imaging of microtubule behavior by spinning disc confocal microscopy, Hela cells were plated and co-transfected with pDest-BIN1-mCherry and α-tubulin-GFP. Time lapse sequence for α-tubulin was acquired at a continuous rate of 1 s with 400 ms exposure per frame. To confirm the similar BIN1 expression pattern, BIN1 images were taken both before and after tubulin time lapse sequence. The tubulin-GFP particle paths were manually traced and analyzed for travel velocity and pause event in the time sequence using MTrackJ Plugin in ImageJ. “Microtubule at BIN1” is considered when the microtubule tip is within 0.2 µm of the closet BIN1 edge.

For calcium imaging in neonatal mouse cardiomyocytes, cardiomyocytes were loaded with a cell permeable calcium dye 4-AM in calcium-free HBSS (Gibco) for 15 min and imaged in regular HBSS (Gibco) with a 20× TIRF objective with a wide-field epifluorescence microscopy. Live images were captured by a high-sensitive Cascade II 512 camera at a frame rate of 64 ms for 20 s.

### Electron Microscopy

For electron microscopy membrane ultrastructure, cells were fixed in Karnovsky's fixative (1% paraformaldehyde / 3% Glutaraldehyde in 0.1 M Sodium cacodylate buffer, pH 7.4) at room temperature for 30 min before being stored at 4°C. The method for the membrane ultrastructure study was previously described [47],[48]. Briefly, the fixed cells were then post-fixed in OsO4 (2% OsO4 + 1.5% potassium ferrocyanide, Sigma) and stained en bloc with 1% tannic acid (Sigma), uranyl acetate (EM Science) before being dehydrated in ethanol, cleared in propyline oxide, and embedded in eponate 12 (Ted Pella Co.). Finally, cells were sectioned and stained with uranyl acetate and Reynold's Lead to enhance contrast and were examined under Philips Tecnai 10 electron microscope (Eidhoven).

For immunolabeling, mouse cardiomyocyte suspension was fixed in 2% paraformaldehyde / 0.1% glutaraldehyde in 0.1 M cacodylate buffer pH7.4 at room temperature for ∼2–3 h. An established procedure [49],[50] was used for immunogold labeling of mouse cardiomyocytes. Briefly, the fixed samples were cryoprotected with PVP/sucrose (20% polyvinyl pyrrolidone [Sigma] in 2.3 M sucrose) overnight and frozen in liquid nitrogen before being cut into thin sections with Leica Ultracut UCT with EMFCS attachment (Leica Microsystems Inc.). Sections were treated with 0.2% glycine, blocked with 2% BSA/gelatin in PBS, pH 7.4, incubated with mouse anti-BIN1 (1∶2, Sigma) and rabbit anti-Cav1.2 (1∶2, Alomone) diluted with blocking solution overnight at room temperature (controls were done with normal mouse serum), and incubated with 10 nm immunogold conjugated anti-mouse (1∶25) and 15 nm immunogold conjugated goat anti-rabbit (1∶50) secondary antibodies for 30 min. The sections were then stained with oxalate uranyl acetate and embedded in 1.5% methyl cellulose (Sigma) and 0.3% aqueous uranyl acetate (Ted Pella Inc.). Colocalization between Cav1.2 and BIN1 was examined in a Philips Tecnai 10 electron microscope (Eidhoven).

### Ethics Statement

With the approval of the University of California–San Francisco (UCSF) Committee for Human Research, we obtained tissue from organ donors whose hearts were not transplanted. The California Transplant Donor Network (CTDN) provided the unused donor hearts and obtained informed consent for their use from the next of kin. All the mouse work was approved by UCSF Committee for Animal Research. All procedures were in accordance with UCSF animal research and care protocols.

### Human Tissue Collection and Cardiomyocytes Isolation

After immediate perfusion with cold cardioplegia, full-thickness samples from left ventricular free wall were cleaned rapidly of all epicardial fat and snap frozen into liquid nitrogen for later protein and mRNA analysis. More sections were embedded in OCT medium and frozen in liquid N_2_-chilled isopentane for immunohistochemistry. For cardiomyocytes isolation, ventricular free wall samples were cut into ∼1 mm^3^ sections for digestion with pre-warmed collagenase II (2 mg/ml, Worthington) at 37°C in calcium-free KHB solution (134 mM NaCl, 11 mM Glucose, 10 mM Hepes, 4 mM KCl, 1.2 mM MgSO_4_, 1.2 mM Na_2_HPO_4_, 10 mM BDM, 0.5 mg/ml BSA, Ph 7.4) [51] with modification of a previously reported method [52]. Dissociated cardiomyocytes were allowed to attach to laminin-precoated glass coverslips before fixation for immunocytochemistry.

### Isolation and Culture of Adult Mouse Cardiomyocytes

Mouse ventricular myocytes were isolated from male adult C6/Black mouse (∼8–12 wk; Charles River) after dissociation with collagenase II (2 mg/ml, Worthington) with a previously described method [53]. For surface biotinylation experiments, cardiomyocytes were attached to laminin-precoated culture dishes and cultured in primary cardiomyocyte medium (ScienCell) in 37°C and 5% CO2 incubator. The cells were treated with vehicle (DMSO, 1∶2000) overnight (16 h) before the replacement with control medium (containing DMSO, 1∶2000) or medium containing 20 µM dynasore with or without 30 µM nocodazole for 2 h. For 18 h nocodazole treatment, cardiomyocytes were cultured in medium containing 30 µM nocodazole overnight (16 h) before the replacement of medium containing dynasore + nocodazole for another 2 h.

### Isolation and Differentiation of Mouse Cardiomyocytes

Timed pregnant mice were ordered from Charles River at E16-17. Primary mouse neonatal cardiomyocytes were isolated from p3/4 C57BL/6 mice and maintained in F12/DMEM 50/50 (Invitrogen) supplemented with 2% FBS, Insulin-transferrin-sodium selenite media supplement, 10 µM 5-Bromo-2′-deocyuridine, 20 µM Cytosine β-D-arabinofuranoside (Sigma), and 100 µg/ml Primocin (Amaxa). Cells were maintained in a humidified atmosphere of 5% CO_2_ at 37°C. Cardiomyocytes were allowed for differentiation in culture for about a week before surface biotinylation and calcium-imaging experiments. After 3 to 4 d in culture, the cells were transfected with 125 nM control or BIN1 siRNA (Dharmacon), which was repeated 24 h later. Three days after the first dose of siRNA, surface biotinylation experiments and calcium imaging were studied in these cells.

### Surface Biotinylation of Cav1.2 and NCX1

After treatment, the cells were quickly washed and incubated with ice-cold 1 mg/ml High Capacity Neutraavidin Agarose Resin (Pierce) for 25 min. After 2 × 5 min quenching of unbound biotin with 100 µM glycine, cells were washed and lysed in RIPA buffer (50 mM Tris pH 7.4, 150 mM NaCl, 1 mM EDTA, 1% Triton X-100, 1% sodium deoxycholate, 2 mM NaF, 200 µM Na_3_VO_4_) supplemented with Complete Mini protease inhibitor cocktail (Roche). Total protein concentrations were determined and normalized between samples. The lysates were then incubated with prewashed NeutrAvidin coated beads at 4°C overnight. After washes, bound surface proteins were eluted and boiled, separated on NuPage gels (Invitrogen), and probed with rabbit anti-Cav1.2 antibody (Alomone) and mouse anti-NCX1 antibody (Abcam). Similar expression levels of BIN1 and BIN1-BAR* were confirmed by Western blot in the total cellular lysates. For quantitation, the amount of surface Cav1.2 or NCX1 was normalized to input and compared among different groups.

### Co-Immunoprecipitation

HeLa cells were cotransfected with human Cav1.2 along with regulatory β2b and α2δ1 subunits and BIN1-V5, harvested, and lysed in 1% Triton X-100 Co-IP buffer (50 mM Tris pH 7.5, 150 mM NaCl, 2 mM EDTA, 2 mM EGTA, 1 mM DTT, 1 mM NaF, 100 µM Na_3_VO_4_, 1% Triton X-100) supplemented with Complete Mini protease inhibitor cocktail. The lysate was then incubated with either mouse anti-V5 antibody (2 µg) or equal amount of non-specific mouse IgG for 2 h before pulldown with rec-protein-G-Sepharose (Invitrogen) for 1 h. Material bound to washed beads was eluted, boiled, separated, and probed with rabbit antibodies against Cav1.2 (Alomone) or V5 (Sigma).

### Signal Processing and Statistical Analysis

For spatial periodicity analysis in the cardiomyocytes, the fluorescence intensity profiles were generated by ImageJ. The frequency domain power spectrum of cardiomyocyte subsections were generated in Matlab using FFT conversion. Next, the power spectrum over spatial distance (1/frequency) was averaged from five cardiomyocytes and presented in Figure 1. For T-tubule Cav1.2 signal, intensity at each peak (corresponding to T-tubules) was analyzed using the fluorescence intensity profiles generated by ImageJ and Matlab. For quantitation of cell peripheral Cav1.2, three-dimensional cross-section projection of cardiomyocytes were generated, and fluorescence intensity within 2 µm of cell surface was analyzed using ImageJ. In addition, a previously reported method [54] using PSC Colocalization plug-in in ImageJ was used for colocalization analysis between BIN1 and Cav1.2. For all other statistical analysis, paired or unpaired two-tail Student's *t* test was performed using Prism 5 (GraphPad) software.

## Supporting Information

Figure S1 **BIN1 is expressed at both T-tubules and nuclei in cardiomyocytes Confocal images (60×) of both human (top) and mouse (bottom) cardiomyocytes.** The cells were fixed and stained with mouse anti-BIN1. DAPI was used to label nuclei. BIN1 is localized at both nuclei and T-tubules (scale bar: 10 µm).
(0.22 MB PDF)

Figure S2 **Cx43 distribution is different from Cav1.2 in cardiomyocytes.** Confocal image (100×) of adult mouse cardiomyocytes. The cells were fixed and stained with mouse anti-Cav1.2 or rabbit anti-Cx43. Three-dimensional volume views of Cav1.2 and Cx43 distribution are reconstructed from a stack of 100× confocal image frames acquired at a z-step of 0.1 µm (first column). Two-dimensional frames of Cav1.2 and Cx43 are shown in the second column. Cardiomyocyte fluorescence intensity profiles along 30 µm of the longitudinal axis are presented in the third column. The bottom panel is the power spectrum over spatial distance for Cx43 averaged from five cardiomyocytes, which indicate that intercalated disc localized Cx43 distribution does not have a similar pattern of Cav1.2 (see Figure 1) (scale bar: 10 µm).
(0.26 MB PDF)

Figure S3 **Whole cell view of BIN1 and Cav1.2 in cardiomyocytes.** Confocal images (60×) of both human (top) and mouse (bottom) cardiomyocytes. Co-staining with mouse anti-BIN1 (green) and rabbit anti-Cav1.2 (red) indicates colocalization of BIN1 and Cav1.2 (scale bar: 10 µm).
(0.25 MB PDF)

Figure S4 **Cx43 does not colocalize with Cav1.2 in cardiomyocytes.** In isolated adult mouse cardiomyocytes, co-staining with Cx43 (red) and Cav1.2 (green) does not indicate colocalization of Cx43 and Cav1.2 (scale bar: 5 µm). Pearson colocalization coefficient and scatter plot reveal no significant colocalization between Cx43 and Cav1.2.
(0.26 MB PDF)

Figure S5 **Microtubule-dependent delivery of Cav1.2. Top: Western blot indicates total cellular protein content of Cav1.2 is not changed by nocodazole.** Confocal images (100×) of mouse cardiomyocytes subjected to control or nocodazole treatment. Staining with rabbit anti-Cav1.2 indicates reduction of Cav1.2 at both general cell periphery as well as along T-tubules (scale bar: 10 µm). Quantitative data are presented in the bottom panel (* *p*<0.05, Student's *t* test).
(0.32 MB PDF)

Figure S6 **Microtubule-dependent forward trafficking of Cav1.2 in HL-1 cells.** Surface biotinylation of endogenous Cav1.2 in cultured HL-1 cells. Nocodazole (30 µM overnight) reduces surface Cav1.2 expression in the presence of an endocytosis inhibitor dynasore (80 µM). Western blot of one representative experiment is shown in the top panel. Quantification data of the Cav1.2 surface expression level summarized from three separate experiments are presented in bar graph shown in the bottom panel (** *p*<0.01, Student's *t* test).
(0.17 MB PDF)

Figure S7 **BIN1 fails to cause surface expression of NCX1 in HL-1 cells.** Surface biotinylation of endogenous Cav1.2 and NCX1 in cultured HL-1 cells transfected with BIN1-BAR* and full-length BIN1. Western blot of one representative experiment is shown in the left panel. Quantification of the Cav1.2 and NCX1 surface expression levels are summarized and presented in bar graph shown in the right panel. Compared with BIN1-BAR*, full-length BIN1 increases surface expression of Cav1.2 but not NCX1 (** *p*<0.01, Student's *t* test).
(0.22 MB PDF)

Figure S8 **Differentiated postnatal mouse cardiomyocytes express BIN1 and have T-tubules.** (A) Confocal images of 1-wk differentiated cardiomyocytes isolated from P3/4 postnatal mice co-stained with mouse anti-BIN1 (green) and rabbit anti-Cav1.2 display T-tubule localization pattern. (B) Quantitative rt-PCR data indicate postnatal mouse heart tissue have a similar expression level of BIN1 compared to young adult heart (8 wk).
(0.22 MB PDF)

Video S1 **Dynamic microtubules associate with BIN1 structures.** Live-cell imaging in HeLa cells transfected with BIN1-mCherry with α-tubulin-GFP. The movie is a 2 min capture period of images acquired at 1 s interval for α-tubulin-GFP with 400 ms exposure per frame. The α-tubulin-GFP sequence is then merged with the BIN1-mCherry frame. Note microtubules (green) appear to tether at BIN1 structures (red). When not interacting with BIN1, microtubules travel rapidly.
(4.32 MB AVI)

## Abbreviations

Cav1.2: L-type calcium channels
CHF: congestive heart failure
CICR: calcium-induced calcium release
Cx43: Connexin43
EC: excitation-contraction
NCX1: sodium calcium exchanger 1
SR: sarcoplasmic reticulum
TIRF: total internal reflection fluorescence
