# Supplementary figures and images for: BIN1 Localizes the L-Type Calcium Channel to Cardiac T-Tubules
Source: PLoS Biol. 2010 Feb 16;8(2):e1000312. doi: 10.1371/journal.pbio.1000312 (PMC2821894; doi:10.1371/journal.pbio.1000312)

## Human Cardiomyocytes

**BIN1**

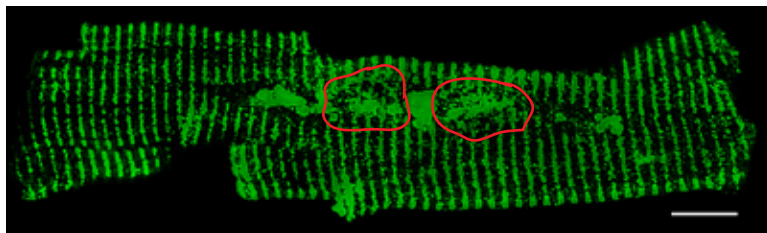

**DAPI**

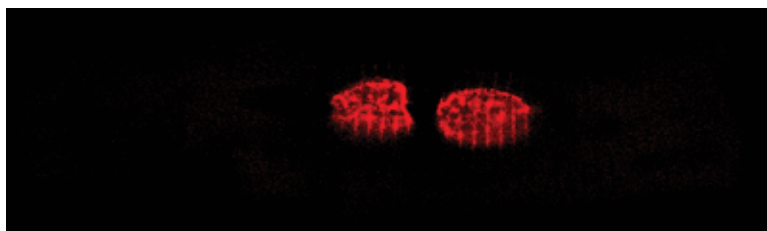

**Merged**

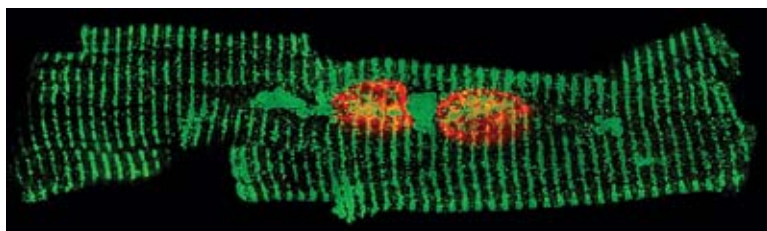

## Mouse Cardiomyocytes

**BIN1**

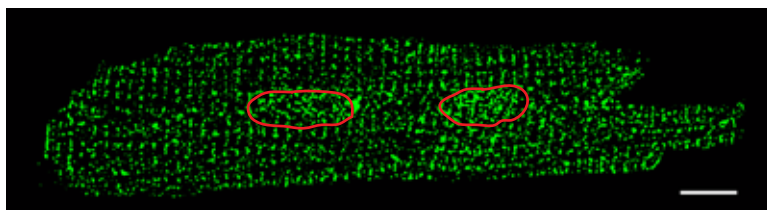

**DAPI**

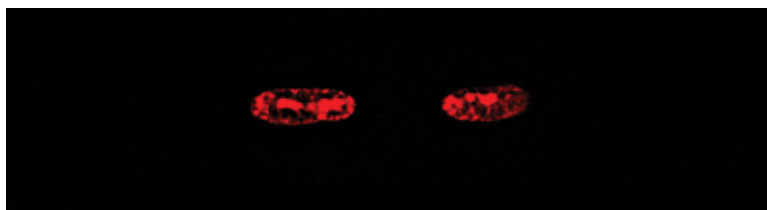

**Merged**

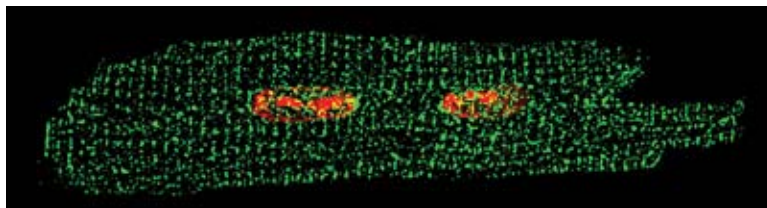

Supplement: Figure S1 — BIN1 is expressed at both T-tubules and nuclei in cardiomyocytes Confocal images (60×) of both human (top) and mouse (bottom) cardiomyocytes. The cells were fixed and stained with mouse anti-BIN1. DAPI was used to label nuclei. BIN1 is localized at both nuclei and T-tubules (scale bar: 10 µm). (0.22 MB PDF) [file pbio.1000312.s001.pdf]

## Human Cardiomyocytes

**BIN1**

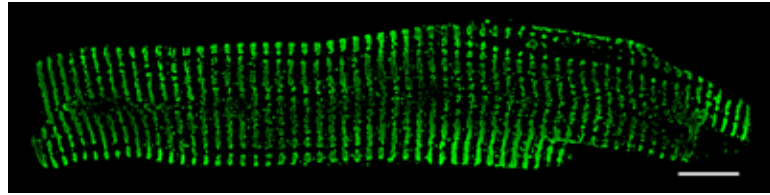

**Cav 1.2**

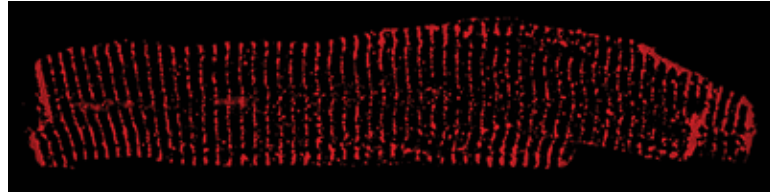

**Merged**

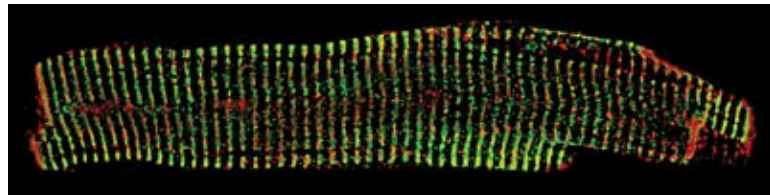

## Mouse Cardiomyocytes

**BIN1**

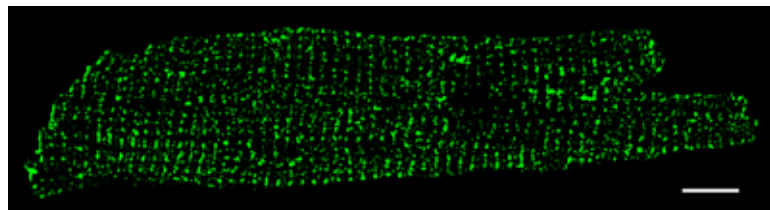

**Cav 1.2**

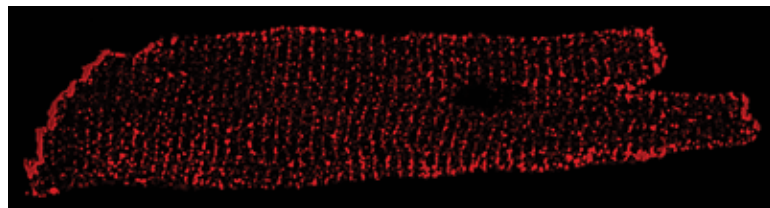

**Merged**

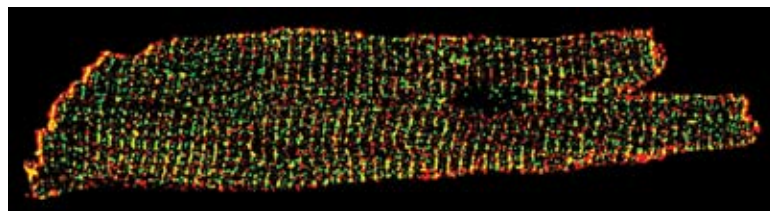

Supplement: Figure S3 — Whole cell view of BIN1 and Cav1.2 in cardiomyocytes. Confocal images (60×) of both human (top) and mouse (bottom) cardiomyocytes. Co-staining with mouse anti-BIN1 (green) and rabbit anti-Cav1.2 (red) indicates colocalization of BIN1 and Cav1.2 (scale bar: 10 µm). (0.25 MB PDF) [file pbio.1000312.s003.pdf]

# Mouse Cardiomyocytes

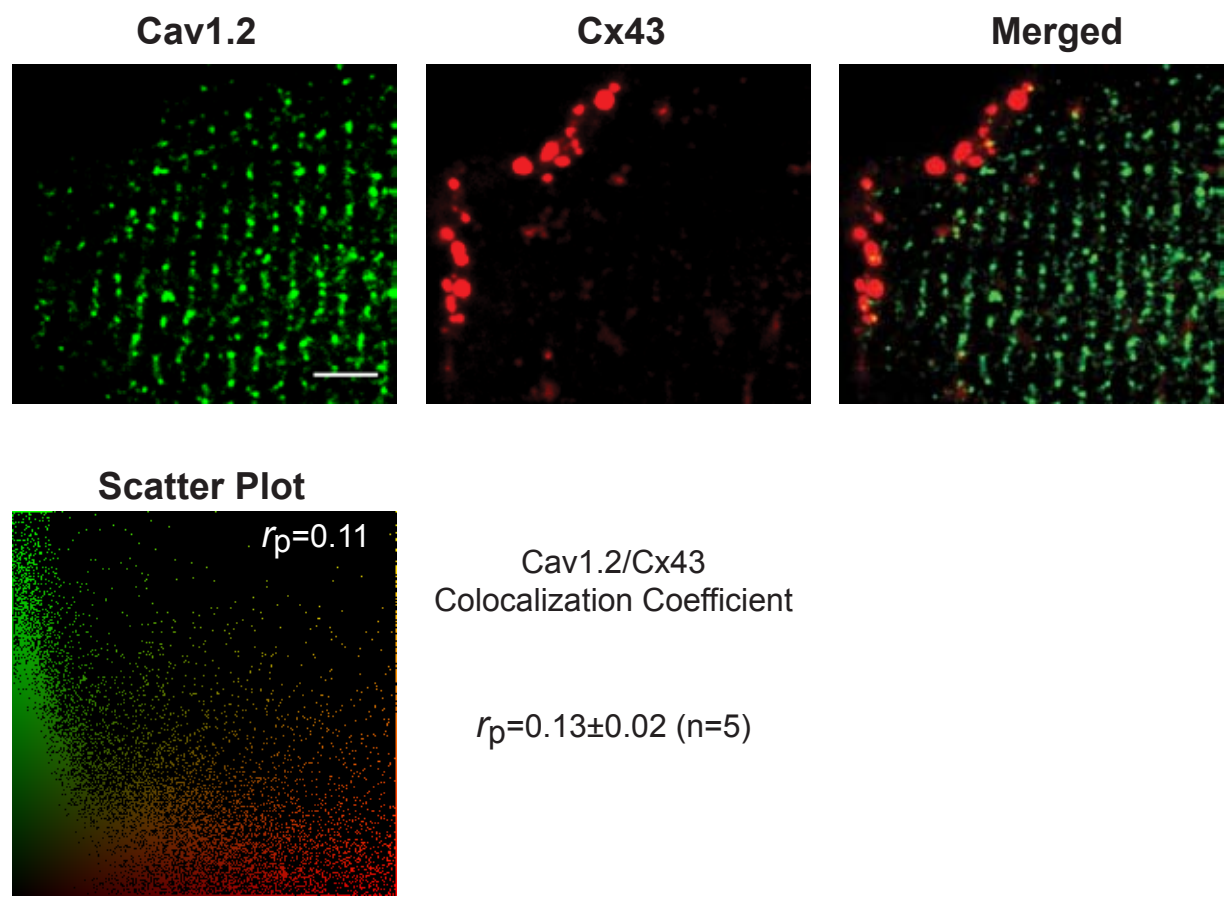

Scatter Plot

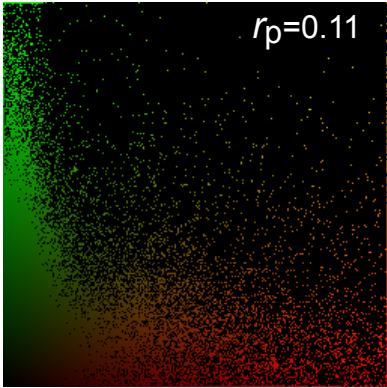

$r_p=0.11$

Cav1.2/Cx43  
Colocalization Coefficient

$r_p=0.13\pm0.02$  (n=5)

Supplement: Figure S4 — Cx43 does not colocalize with Cav1.2 in cardiomyocytes. In isolated adult mouse cardiomyocytes, co-staining with Cx43 (red) and Cav1.2 (green) does not indicate colocalization of Cx43 and Cav1.2 (scale bar: 5 µm). Pearson colocalization coefficient and scatter plot reveal no significant colocalization between Cx43 and Cav1.2. (0.26 MB PDF) [file pbio.1000312.s004.pdf]

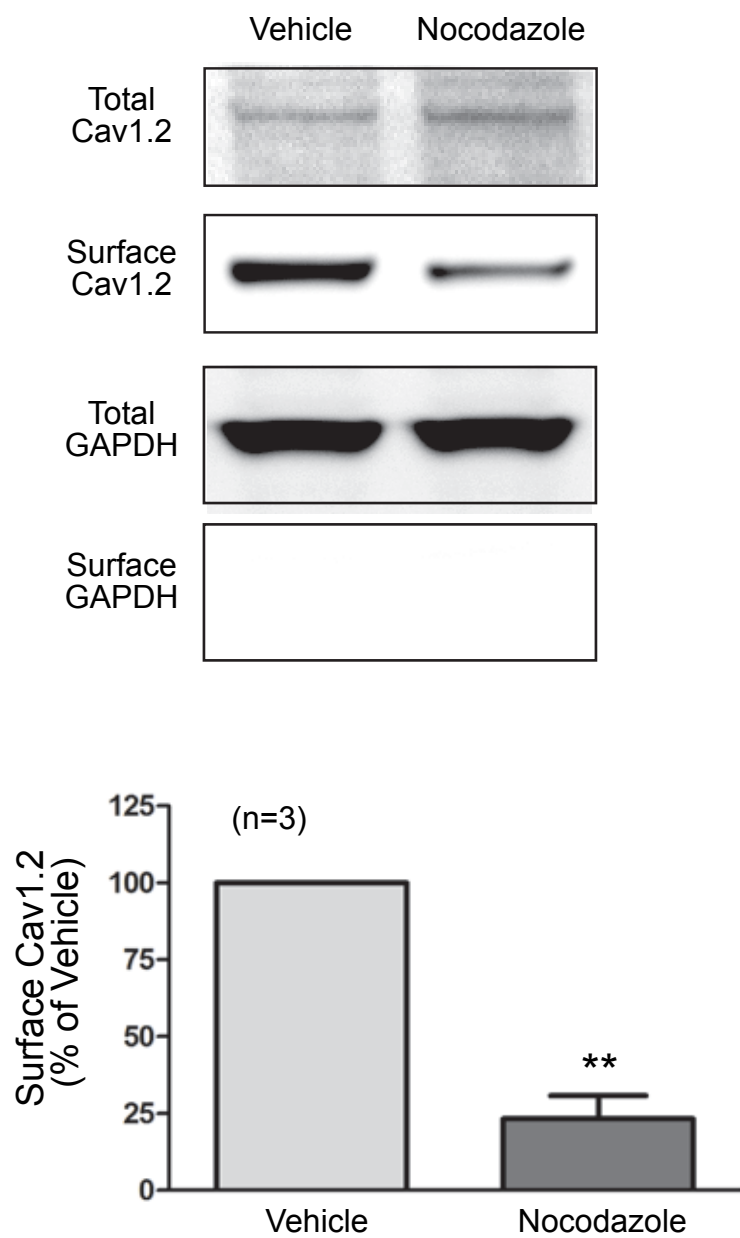

**Supplemental Figure S6**

Supplement: Figure S6 — Microtubule-dependent forward trafficking of Cav1.2 in HL-1 cells. Surface biotinylation of endogenous Cav1.2 in cultured HL-1 cells. Nocodazole (30 µM overnight) reduces surface Cav1.2 expression in the presence of an endocytosis inhibitor dynasore (80 µM). Western blot of one representative experiment is shown in the top panel. Quantification data of the Cav1.2 surface expression level summarized from three separate experiments are presented in bar graph shown in the bottom panel (** p<0.01, Student's t test). (0.17 MB PDF) [file pbio.1000312.s006.pdf]

# BIN1 fails to induce surface expression of NCX1 in HL1 cells

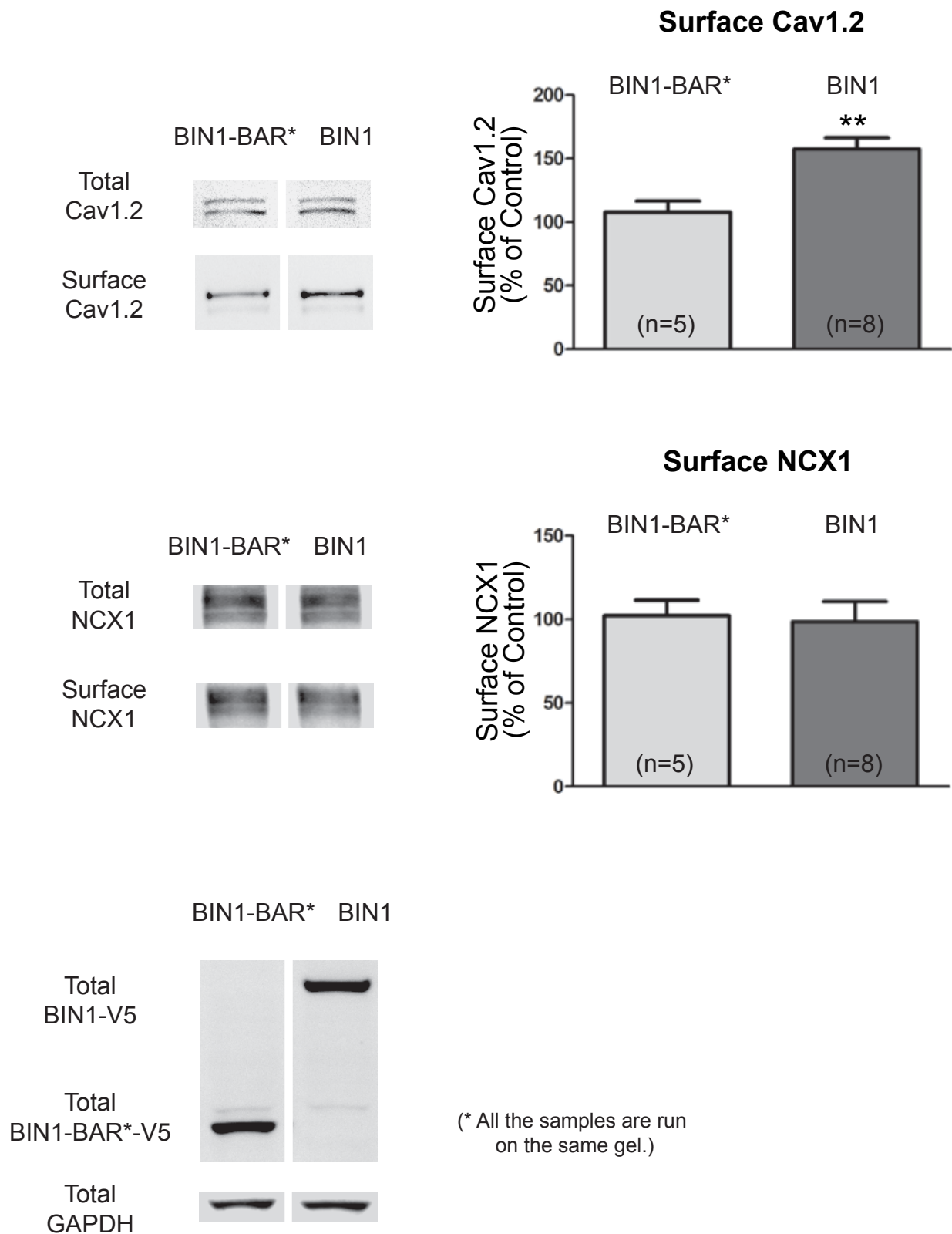

**Supplemental Figure S7**

Supplement: Figure S7 — BIN1 fails to cause surface expression of NCX1 in HL-1 cells. Surface biotinylation of endogenous Cav1.2 and NCX1 in cultured HL-1 cells transfected with BIN1-BAR* and full-length BIN1. Western blot of one representative experiment is shown in the left panel. Quantification of the Cav1.2 and NCX1 surface expression levels are summarized and presented in bar graph shown in the right panel. Compared with BIN1-BAR*, full-length BIN1 increases surface expression of Cav1.2 but not NCX1 (** p<0.01, Student's t test). (0.22 MB PDF) [file pbio.1000312.s007.pdf]
