# Supplementary material for: BIN1 Localizes the L-Type Calcium Channel to Cardiac T-Tubules
Source: PLoS Biol. 2010 Feb 16;8(2):e1000312. doi: 10.1371/journal.pbio.1000312 (PMC2821894; doi:10.1371/journal.pbio.1000312)
Supplement: Figure S2 — Cx43 distribution is different from Cav1.2 in cardiomyocytes. Confocal image (100×) of adult mouse cardiomyocytes. The cells were fixed and stained with mouse anti-Cav1.2 or rabbit anti-Cx43. Three-dimensional volume views of Cav1.2 and Cx43 distribution are reconstructed from a stack of 100× confocal image frames acquired at a z-step of 0.1 µm (first column). Two-dimensional frames of Cav1.2 and Cx43 are shown in the second column. Cardiomyocyte fluorescence intensity profiles along 30 µm of the longitudinal axis are presented in the third column. The bottom panel is the power spectrum over spatial distance for Cx43 averaged from five cardiomyocytes, which indicate that intercalated disc localized Cx43 distribution does not have a similar pattern of Cav1.2 (see Figure 1) (scale bar: 10 µm). (0.26 MB PDF) [file pbio.1000312.s002.pdf]

Mouse Cardiomyocytes

Cav1.2

3D Volume

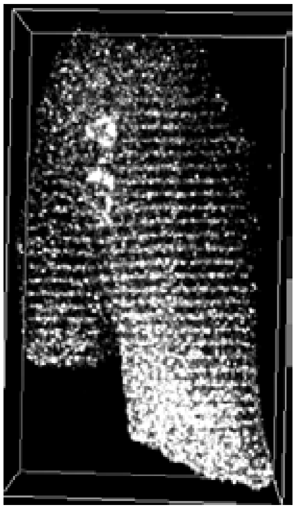

2D Frame

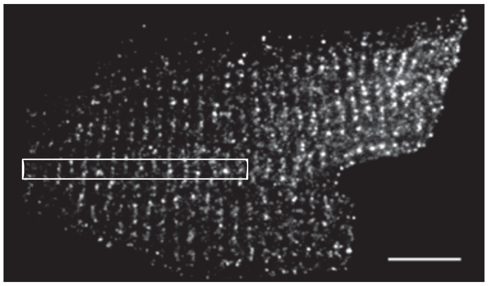

Spatial Periodicity

Fluorescence intensity along longitudinal axis

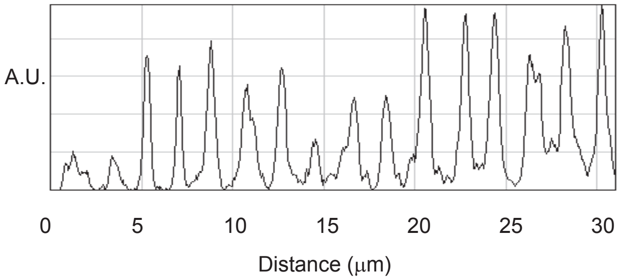

Connexin43

3D Volume

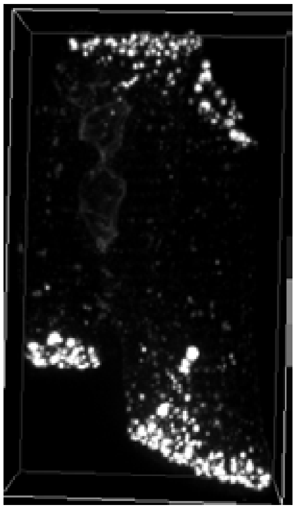

2D Frame

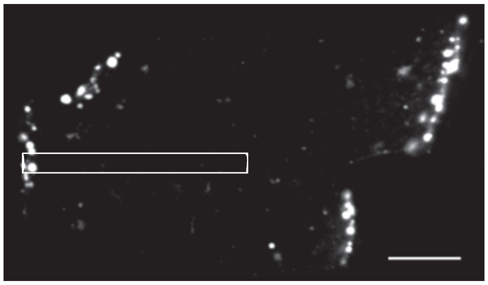

Spatial Periodicity

Fluorescence intensity along longitudinal axis

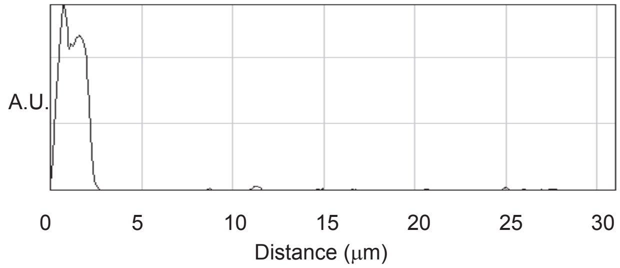

Power Spectrum (n=5)

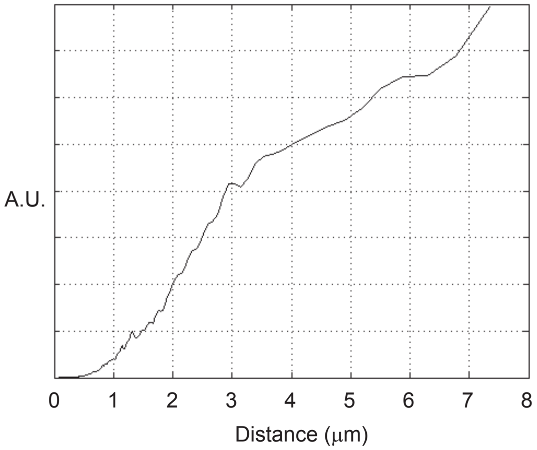

Supplemental Figure S2
