# Supplementary material for: BIN1 Localizes the L-Type Calcium Channel to Cardiac T-Tubules
Source: PLoS Biol. 2010 Feb 16;8(2):e1000312. doi: 10.1371/journal.pbio.1000312 (PMC2821894; doi:10.1371/journal.pbio.1000312)
Supplement: Figure S5 — Microtubule-dependent delivery of Cav1.2. Top: Western blot indicates total cellular protein content of Cav1.2 is not changed by nocodazole. Confocal images (100×) of mouse cardiomyocytes subjected to control or nocodazole treatment. Staining with rabbit anti-Cav1.2 indicates reduction of Cav1.2 at both general cell periphery as well as along T-tubules (scale bar: 10 µm). Quantitative data are presented in the bottom panel (* p<0.05, Student's t test). (0.32 MB PDF) [file pbio.1000312.s005.pdf]

Adult mouse cardiomyocytes

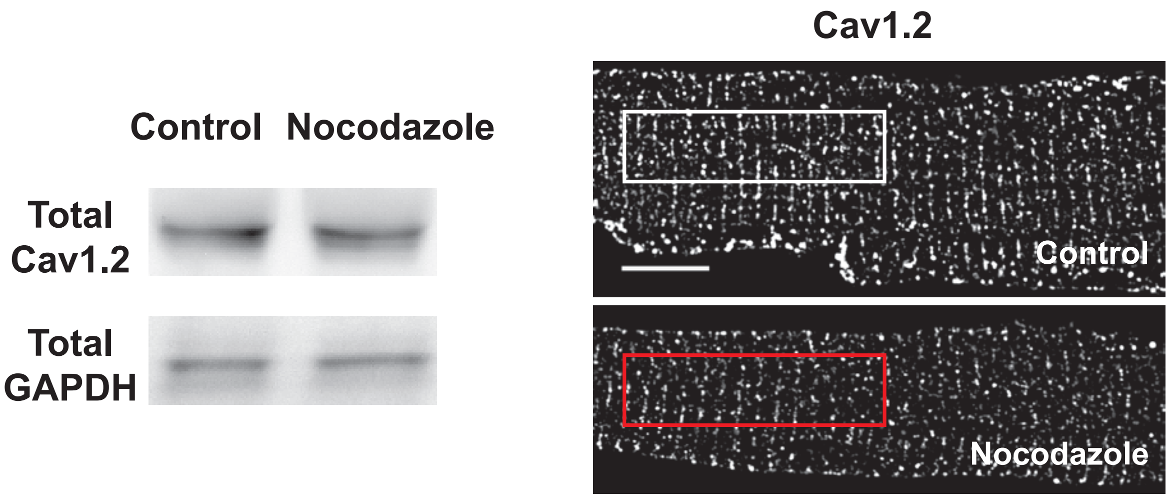

Nocodazole decreases general cell peripheral Cav1.2

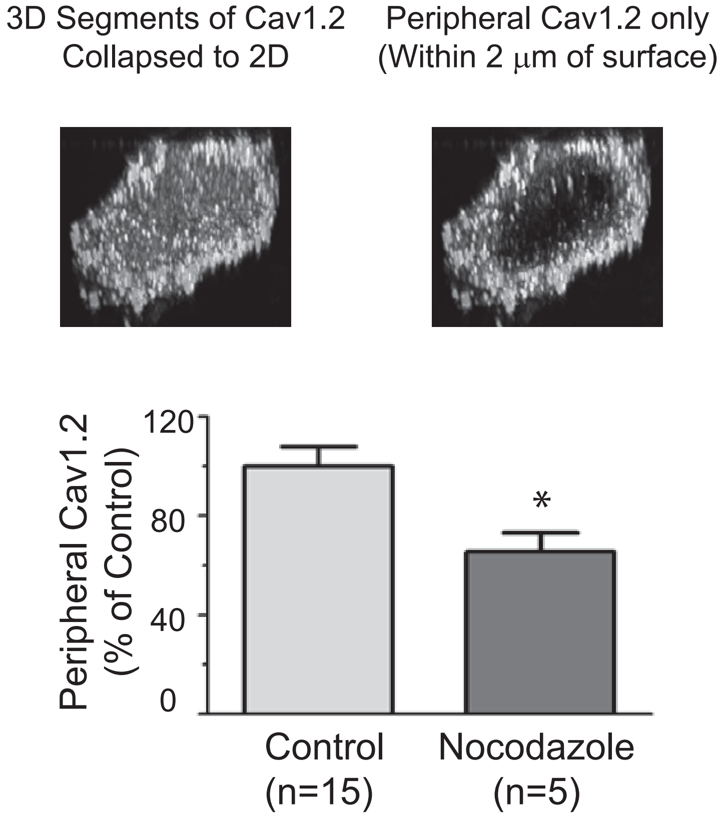

Nocodazole decreases T-tubule Cav1.2

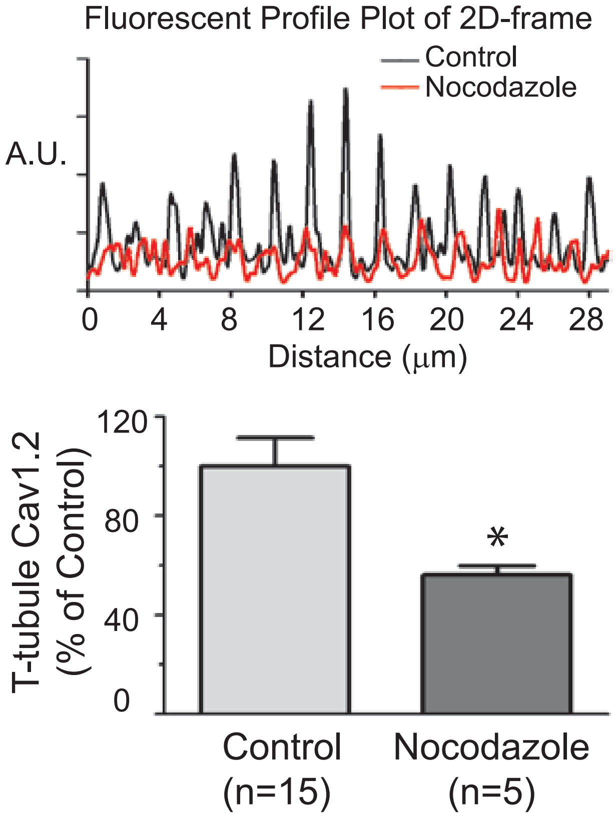

Supplemental Figure S5
