# Supplementary material for: BIN1 Localizes the L-Type Calcium Channel to Cardiac T-Tubules
Source: PLoS Biol. 2010 Feb 16;8(2):e1000312. doi: 10.1371/journal.pbio.1000312 (PMC2821894; doi:10.1371/journal.pbio.1000312)
Supplement: Figure S8 — Differentiated postnatal mouse cardiomyocytes express BIN1 and have T-tubules. (A) Confocal images of 1-wk differentiated cardiomyocytes isolated from P3/4 postnatal mice co-stained with mouse anti-BIN1 (green) and rabbit anti-Cav1.2 display T-tubule localization pattern. (B) Quantitative rt-PCR data indicate postnatal mouse heart tissue have a similar expression level of BIN1 compared to young adult heart (8 wk). (0.22 MB PDF) [file pbio.1000312.s008.pdf]

Differentiated postnatal mouse cardiomyocytes express BIN1 at T-tubules

A Differentiated CMs have a T-Tubule staining pattern of BIN1 and Cav1.2

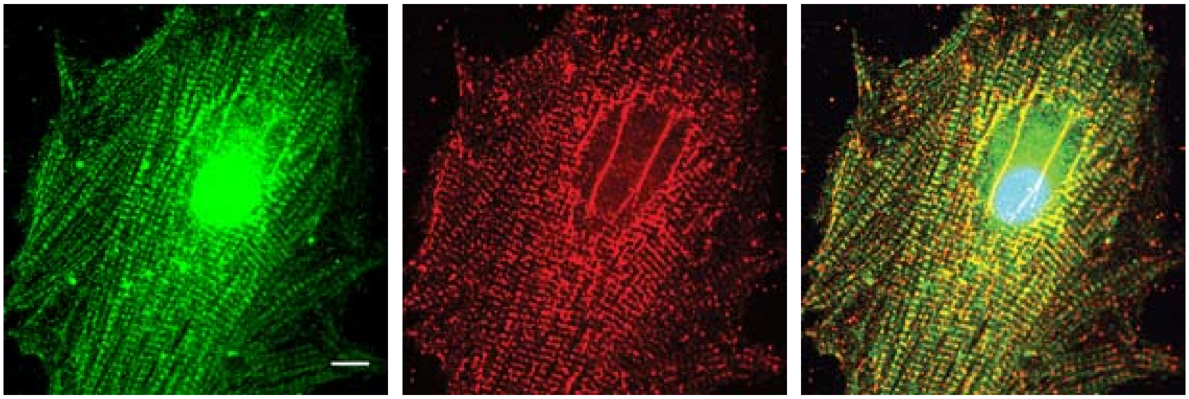

B Postnatal mouse heart tissue express BIN1 similarly to Adult CM

Rt-PCR

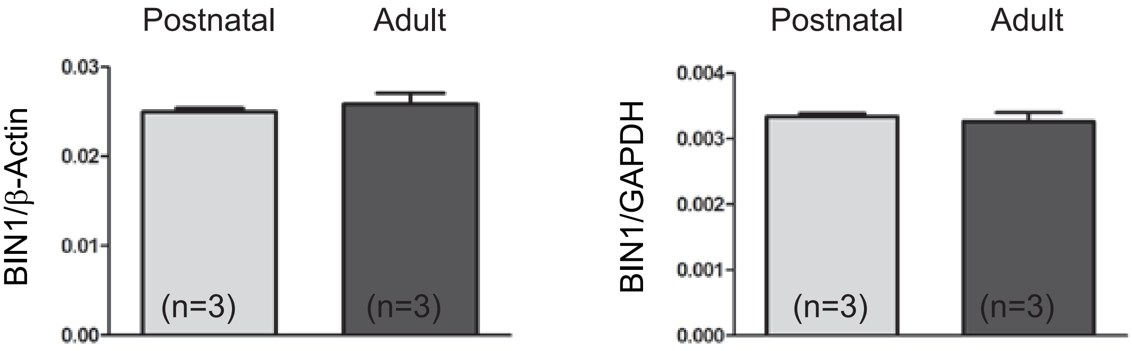

Supplemental Figure S8
